# Selection drives convergent gene expression changes during transitions to co-sexuality in haploid sexual systems

**DOI:** 10.1038/s41559-022-01692-4

**Published:** 2022-03-21

**Authors:** Guillaume G. Cossard, Olivier Godfroy, Zofia Nehr, Corinne Cruaud, J. Mark Cock, Agnieszka P. Lipinska, Susana M. Coelho

**Affiliations:** 1 Sorbonne Université, UPMC Univ Paris 06, CNRS, Integrative Biology of Marine Models, Station Biologique de Roscoff, CS, Roscoff, France; 2 Max Plank Institute for Biology Tübingen, Tübingen, Germany; 3 Genoscope, Institut de Biologie François Jacob, CEA, Université Paris-Saclay, Evry, France

**Keywords:** Evolutionary genetics, Marine biology

## Abstract

Co-sexuality has evolved repeatedly from unisexual (dioicous) ancestors across a wide range of taxa. However, the molecular changes underpinning this important transition remain unknown, particularly in organisms with haploid sexual systems such as bryophytes, red algae and brown algae. Here we explore four independent events of emergence of co-sexuality from unisexual ancestors in brown algal clades to examine the nature, evolution and degree of convergence of gene expression changes that accompany the breakdown of dioicy. The amounts of male versus female phenotypic differences in dioicous species were not correlated with the extent of sex-biased gene expression, in stark contrast to what is observed in animals. Although sex-biased genes exhibited a high turnover rate during brown alga diversification, some of their predicted functions were conserved across species. Transitions to co-sexuality consistently involved adaptive gene expression shifts and rapid sequence evolution, particularly for male-biased genes. Gene expression in co-sexual species was more similar to that in females rather than males of related dioicous species, suggesting that co-sexuality may have arisen from ancestral females. Finally, extensive convergent gene expression changes, driven by selection, were associated with the transition to co-sexuality. Together, our observations provide insights on how co-sexual systems arise from ancestral, haploid UV sexual systems.

## Abstract (web summary)

Studying four examples of the transition to co-sexuality in brown algae, the authors show extensive convergent changes in gene expression driven by selection, and greater similarity of co-sexual gene expression profiles to those of ancestral females than to those of ancestral males.

## Main

Eukaryotic organisms exhibit a wide diversity of sexual systems, ranging from separate sexes (referred to as gonochorism in animals and dioecy in plants) to co-sexuality (combined sexes), and several theories have been developed to explain what conditions favour which strategy^1–7^. The evolution of this diversity often involved transitions between sexual systems. For example, separate sexes have evolved from co-sexual ancestors independently many times in several eukaryotic lineages, and the fundamental mechanisms and evolutionary drivers of this important transition have been intensively studied in many organisms (reviewed in refs. ^2,8^). Frequently, organisms with separate sexes display marked sexual dimorphism in a range of morphological, behavioural and physiological traits. Females and males are nevertheless genetically similar except in the sex-specific regions of their sex chromosomes. While sex chromosomes necessarily play a role in the expression differences between sexes, most sex-biased gene expression involves autosomal genes^9–11^. Differences in autosomal gene expression patterns between sexes may be associated with different physiological processes directly linked to the production of male or female gametes (primary sexual dimorphism) or to the consequences of sexual selection and/or sexual specialization (secondary sexual dimorphism) that may occur once separate sexes have evolved^12^.

While the emergence of separate sexes from co-sexual ancestors and the evolution of sexual dimorphism have been thoroughly investigated^11,13–15^, less attention has been devoted to the opposite transition—that is, from separate sexes to co-sexuality. Transitions to co-sexuality have occurred frequently during eukaryotic evolution and are relatively common in animals (for example, refs. ^13,16–20^). In flowering plants, this transition was believed to be rare, but recent studies are increasingly providing evidence that dioecy-to-monoecy transitions may have occurred frequently^21,22^. Evolutionary models intending to decipher the causes of such transitions invoke the sex-allocation theory^5^ and the deterministic fate of genetic modifiers causing the acquisition of an opposite-sex function^23,24^. However, empirical knowledge on the proximate mechanisms and forces driving the shift from separate sexes to co-sexuality remains largely elusive.

Transitions from separate sexes to co-sexuality are also prevalent in eukaryotic lineages other than animals and flowering plants, particularly those that express sex during the haploid stage of their life cycles. In organisms such as bryophytes, liverworts, and green, red and brown algae, male and female sexes are expressed during the haploid (gametophyte) stage^25^. The terms ‘dioicy’ (that is, separate sexes during the haploid phase of the life cycle, as opposed to ‘dioecy’, where separate sexes occur in the diploid phase) and monoicy (that is, co-sexuality during the haploid phase of the life cycle, as opposed to ‘monoecy’, where co-sexuality occurs in the diploid phase) are used to describe the sexual systems of these organisms^26^. Genetic sex determination in dioicous organisms occurs during meiosis (and not at fertilization as in XY and ZW systems)^27^, depending on whether spores inherit a U or V sex chromosome^26,28^. Spores receiving a V chromosome will develop into male multicellular individuals (male gametophytes), and the spores inheriting a U chromosome will grow into females (female gametophytes). Organisms with haploid sex determination may also produce male and female sexual structures in the same (co-sexual) individual (monoicy)^29,30^. Despite the prevalence of haploid sexual systems among eukaryotes, the gene expression changes and evolutionary forces underlying transitions from dioicy to monoicy have remained largely unknown.

In this context, the brown algae represent a particularly attractive group for studies of the evolution of sexual systems and the breakdown of dioicy. The brown algae are a complex multicellular lineage that is part of the stramenopile (or heterokont) supergroup, which also includes diatoms and oomycetes, and they diverged from the Archaeplastida lineage at the time of the eukaryotic crown radiation^31^. Most brown algae have a haplo-diplontic life cycle, with a haploid gametophyte generation alternating with a diploid sporophyte generation. In these brown algae, sexuality is expressed in the haploid generation, with male and female gametes produced either by the same haploid individual (monoicy) or on two separate haploid individuals (dioicy). Dioicy is the prevalent reproductive system^29,32^. This situation contrasts markedly with that described for flowering plants, where only about 6% of extant species have separate sexes, and is more similar to that of bryophytes and liverworts^30^. Dioicous brown algae may exhibit a broad range of levels of sexual dimorphism, both at the level of the gametophytes and with respect to the difference between male and female gamete size^29,32^. While the predicted ancestral state in the brown algae is dioicy, transitions to monoicy have occurred frequently and independently in different clades^32,33^. The independent emergence of monoicous lineages from dioicous ancestors makes this group particularly interesting to examine the genomic consequences and mechanisms underlying the breakdown of dioicy.

**Fig. 1:**
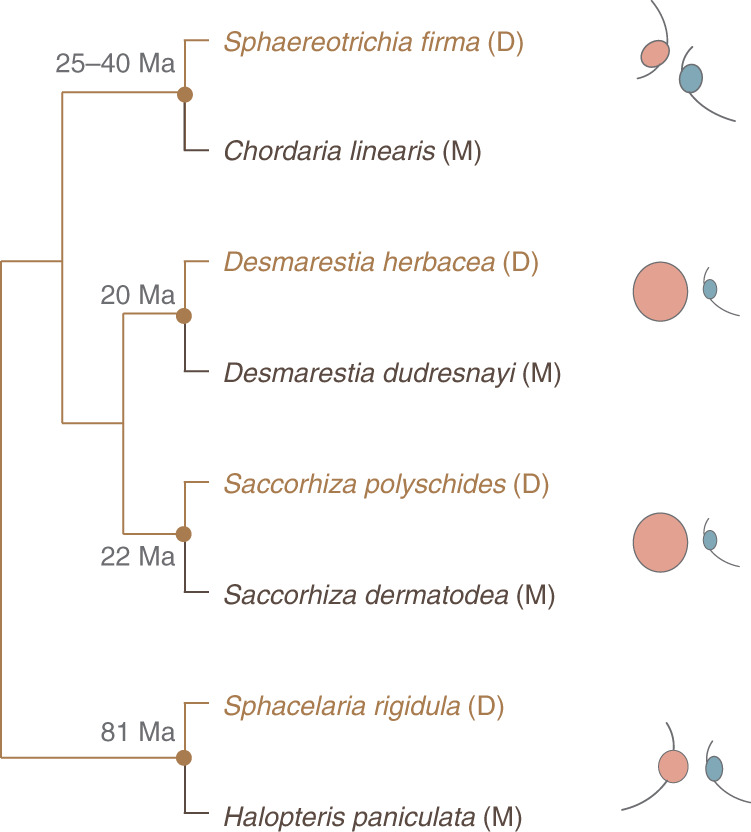
Phylogeny of the eight species of brown algae investigated. The approximate estimated ages of the nodes are based on ref. ^34^ and O. de Clerck (personal communication). A schematic view of typical gamete size differences (female in red and male in blue) for each species pair is presented. Dioicous species (D) are marked in brown and monoicous species (M) in black. Ma, million years ago.

Here we explore multiple, repeated events of loss of dioicy (Fig. 1) to investigate the molecular basis and level of convergence of the shifts to co-sexuality. We test the hypothesis that sexually dimorphic algae might be expected to have more sex-biased genes, and, because dioicy is ancestral, we predicted that similar gene sets would be sex-biased across all the dioicous species. Contrary to our prediction, we demonstrate a lack of correlation between phenotypic sexual dimorphism and gene expression levels among dioicous brown algae. Ancestral state reconstruction indicated high turnover rates of sex-biased genes, yet independently recruited sex-biased genes shared similar functions across the species. To characterize the molecular changes associated with the evolution of monoicy, we then focused on modifications in gene expression patterns of orthologous genes that are specifically or preferentially expressed in haploid males and females of a dioicous species, when they function in a monoicous context. Male-biased genes were particularly characterized by both adaptive expression shifts and faster evolutionary rates associated with the transition to monoicy. Monoicous species displayed expression profiles that were more similar to those of the female of the closely related dioicous species than to those of the male. Finally, we identified a pronounced level of convergent gene expression changes associated with the emergence of co-sexuality, which were probably driven by selection.

## Results

The present study examines sex-biased gene expression in dioicous brown algae and the gene expression changes associated with the transition from dioicy to monoicy. We based our analysis on transcriptomes sequenced from pairs of dioicous–monoicous species in four major clades of brown algae spanning approximately 200 million years of evolution^34^. The transitions are predicted to have occurred at different times in the past (between 20 and 88 million years ago; Fig. 1). Each pair represents an independent transition from dioicy to monoicy. We chose dioicous species with different levels of gamete dimorphism, reflecting the diverse levels of gamete dimorphism occurring across brown algae.

### Sex-biased gene expression in dioicous brown algae

Gene expression patterns in gametophytes of the eight brown algal species were measured by deep sequencing (RNA-seq) of complementary DNA from male, female and co-sexual gametophytes. Transcript abundance (measured as transcripts per million (TPM)) was strongly correlated between biological replicates, with *r*^2^ ranging from 0.89 to 0.99 (Supplementary Table 1). Counts of expressed genes (TPM > 5th-percentile counts across all genes in at least one sample) identified a number of expressed genes that ranged from 13,180 to 27,391 (Supplementary Table 1).

DESeq2 was used to identify genes that were differentially expressed in each of the sexes of the dioicous species^35^. The analysis retained only genes that displayed at least a twofold change in expression level between sexes (fold-change (FC) > 2, *P*_adj_ < 0.05). Note that sex-linked genes (genes located in the sex-specific regions on the V (male) and U (female) sex chromosomes; Methods) were removed from the set of sex-biased genes and thus excluded from further analysis.

**Fig. 2:**
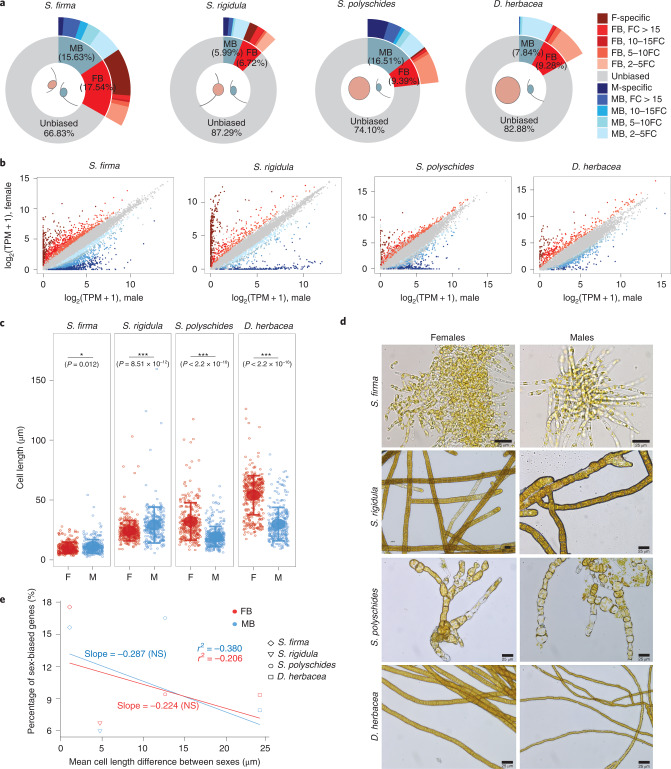
Patterns of sexual dimorphism in dioicous brown algae. **a**, Pie charts representing the fractions of sex-biased genes among expressed genes (FBGs in red and MBGs in blue) in the four dioicous species. The colour gradients represent the intensity of expression FC, from a 2FC difference to more than 15FC. The percentages are calculated on the basis of the total number of expressed genes averaged across sexes. F, female; M, male. **b**, Comparison of gene expression levels, in log_2_(TPM + 1), between males and females within dioicous species. The colour patterns follow the ones used in **a**, except the grey points, which represent unbiased genes that presented an FC > 2. **c**, Scatterplots of the lengths of cells of immature gametophytes of dioicous species. The means (solid points) and standard deviations (whiskers) are plotted per sex per species. The asterisks indicate significant differences between mean cell lengths, tested with two-sided *t*-tests. *0.01 < *P* < 0.05; ***P < 0.001. **d**, Representative micrographs of male and female immature gametophytes viewed under an inverted light microscope for each dioicous species investigated. The micrographs show individual algae, representative of 100–200 individuals grown in petri dishes. **e**, Linear regressions of the fraction of FBGs and MBGs (in red and blue, respectively) among the mean number of expressed genes across both sexes, against the mean difference in cell length recorded between the sexes (in µm), in the four dioicous species investigated. Linear regressions were fitted through the phylogenetic generalized least squares method, implemented in the R package nlme. We report values of adjusted *r*^2^ calculated with analysis of variance. NS, not significant.

All four dioicous brown algae displayed substantial sex-biased gene expression (at least compared with plants and other brown alga^13,15,36^), ranging from 12.71% of the expressed genes in *Sphacelaria* *rigidula* to 33.17% in *Sphaerotrichia* *firma* (Fig. 2a,b and Supplementary Tables 2 and 3). We found similar proportions of male-biased genes (MBGs) and female-biased genes (FBGs) for the majority of the studied species (Fig. 2a,b) except *Saccorhiza* *polyschides*, where MBGs were more abundant than FBGs (16.51% MBGs versus 9.39% FBGs; *χ*^2^ test; *P* < 2.2 × 10^−16^).

### Sex-biased gene expression and phenotypic sexual dimorphism

To investigate the link between sex-biased gene expression and the level of sexual dimorphism, we carried out morphometric measurements of male and female gametophytes complemented with literature searches. These measurements allowed us to quantify the amount of phenotypic dimorphism present in each of the four dioicous species (Supplementary Table 3 and Fig. 2c). In all dioicous species, gamete size dimorphism was coherent with sexual differences in terms of gametophyte cell size (Supplementary Table 3). For example, *Desmarestia* *herbacea* gametophytes presented marked sexual dimorphism in both gamete size and gametophyte cell length, whereas *S. firma* had the least sexual difference in both gametophyte morphology and gamete size (Supplementary Table 4 and Fig. 2c,d).

In animals, sexual differences at the phenotypic level are correlated with levels of sex-biased gene expression^14,37^, but this correlation has not been found in plants^36^. We compared the differences in gametophyte cell size between males and females with the proportion of sex-biased genes in each of the four dioicous brown algal species. We detected no correlation between phenotypic sexual dimorphism (gametophyte cell size) and the number of sex-biased genes (Fig. 2e). For instance, *S. firma* exhibited the highest level of sex-biased gene expression and nonetheless presented the lowest level of phenotypic sexual dimorphism. Taken together, our observations indicate a considerable level of sex-biased gene expression in the four dioicous species studied here, but the level of sex-biased gene expression did not reflect the level of morphological dimorphism between males and females.

### Evolution of sex-biased gene expression in dioicous species

**Extended Data Fig. 1:**
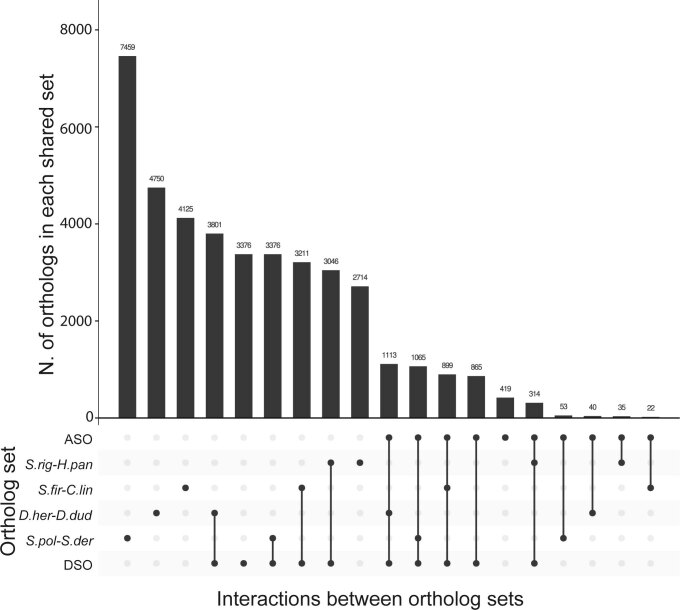
Single copy orthologs gene sets. Representation of intersects across single-copy ortholog gene sets (that is, the four PSO, DSO and ASO) using UpSetR. Bars represent the number of genes in the intersect represented below the histogram. PSO: pairwise single-copy orthologs. DSO: dioicous single-copy orthologs. ASO: All species single-copy orthologs. If we consider for instance the line ‘S.fir-C.lin’, a set of 4125 orthologs are found within PSOs, 3211 are also found in the DSO set, 899 are also members of both the DSO and ASO gene sets and, finally, 22 orthologs are present in the ASO only. Note that the orthofinder analysis was performed independently for DSO and ASO (see methods), therefore some ASOs do not overlap with DSO.

We next investigated how sex-biased gene expression has evolved by comparing the four dioicous brown algal species. Orthofinder identified a total of 14,017 orthogroups (OGs) across the dioicous species, of which 2,098 contained only one gene per species and therefore represented the set of 1:1:1:1 OGs. An additional 2,778 OGs had a single member in each of three of the studied species (that is, the gene was missing in the fourth species). We considered that these 1:1:1:0 OGs, which probably represent single-copy ancestral genes that were lost in one of the species, also provide useful information about the conservation of sex-biased gene expression. Note that the 1:1:1:0 OGs could also represent OGs where one of the genes is missing from one of the genome assemblies, particularly the draft genome assembly for *S. rigidula*. Furthermore, we also included 1,085 OGs with a duplicated gene in a single species (1:1:1:2 OGs) that aligned along more than 60% of their length, resulting in 5,961 dioicous single-copy orthologs (DSOs; Supplementary Table 5 and Extended Data Fig. 1).

**Fig. 3:**
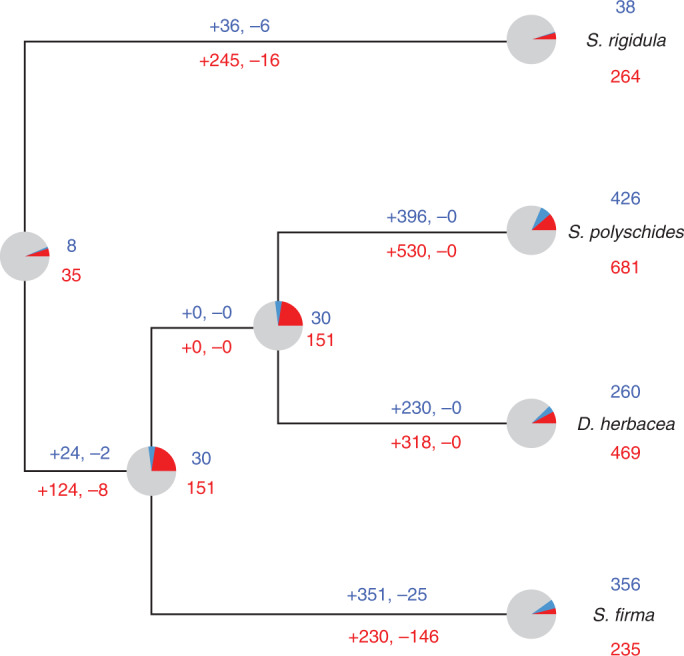
Reconstruction of ancestral sex-biased gene sets across the four dioicous species. The number of inferred sex-biased genes (FBGs in red and MBGs in blue) at ancestral nodes and the inferred gain and loss of sex-biased genes along branches are displayed.

We then used maximum likelihood approaches to infer the ancestral states of sex-biased gene expression across these dioicous species (Fig. 3b). Our analysis identified very few genes that were predicted to be ancestrally sex-biased, with the vast majority having evolved sex bias at some point along the branches. Among the 2,116 sex-biased DSOs in at least one species, only 43 (2.03%) were inferred to be sex-biased in the last common ancestor of the four brown algal species (Fig. 3). Accordingly, no DSOs were consistently sex-biased across the four species (not different from what is expected by chance; exact test multi-set intersection, *P* = 0.506). A total of 139 OGs exhibited a bias in one species that was inconsistent with the direction of bias observed in at least one other species (Supplementary Table 5).

**Extended Data Fig. 2:**
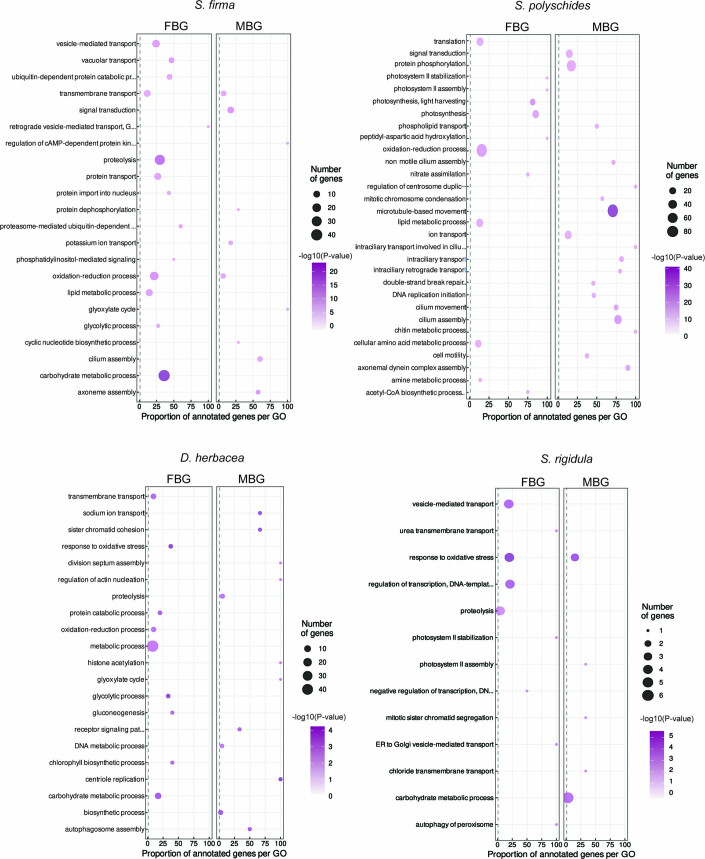
Enriched GO-terms associated with sex-biased genes from each species, tested by Fisher exact tests in TopGO.

Although the above analysis showed that sex-biased genes were not conserved among the four species, we examined whether sex-biased genes in different species were involved in similar functions, by comparing gene ontology (GO) terms of sex-biased genes across species using Blast2GO^38^. We detected significant enrichment of GO terms for biological processes related to ion transport, transmembrane transport and cilia often associated with MBGs across dioicous species. Conversely, the sets of FBGs of all the studied species were enriched for GO terms related to oxidation/reduction (Extended Data Fig. 2 and Supplementary Table 6). Taken together, our results indicate that while sex-biased genes exhibited a high turnover rate during brown alga diversification, some of their predicted functions were conserved across dioicous species.

**Extended Data Fig. 3:**
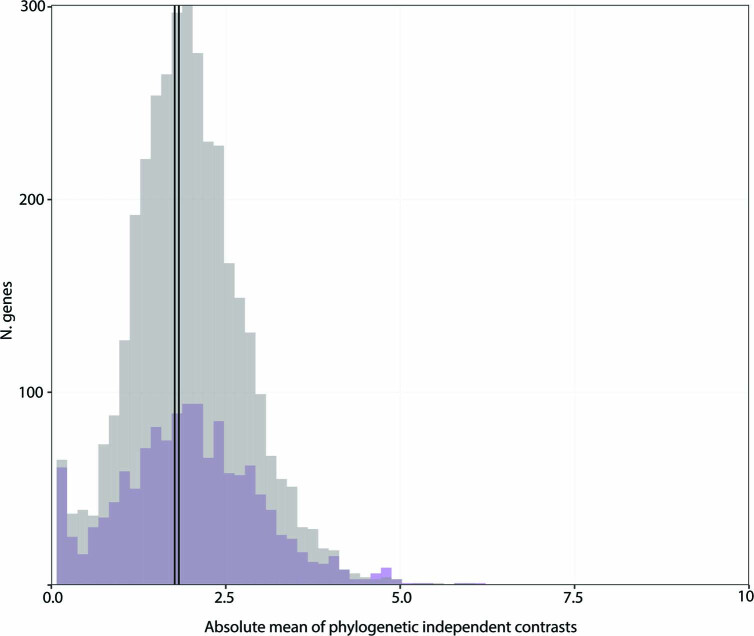
Bar plots representing the mean absolute standardized phylogenetically independent contrasts (PICs) of gene expression, in log_2_(TPM+1) for sex-biased (in purple) and unbiased genes (in grey). We recorded a difference of means of 0.06 between the two groups (Mann-Whitney ranked test *P* = 0.0495).

We also asked whether sex-biased gene expression emerged in dioicous species as a result of random expression evolution under low selective pressure for non-pleiotropic genes^36^ or rather as a consequence of sexual selection. To distinguish between these two possibilities, we computed phylogenetic independent contrasts (PICs) of sex-biased genes across species in which those genes are not sex-biased versus unbiased genes (Extended Data Fig. 3). We found that PICs differed slightly between unbiased genes and orthologs of sex-biased genes in species in which those genes are not sex-biased (Mann–Whitney rank test, *P* = 0.0495). This result indicates that genes that evolved sex bias may have done so because they already experienced low constraints on their expression levels, possibly due to lower pleiotropic expression patterns^36,39^, although we cannot exclude the possibility that sexual selection was also involved in the emergence of sex-biased gene expression in brown algae.

### Sex-biased gene expression fate during transitions to monoicy

**Fig. 4:**
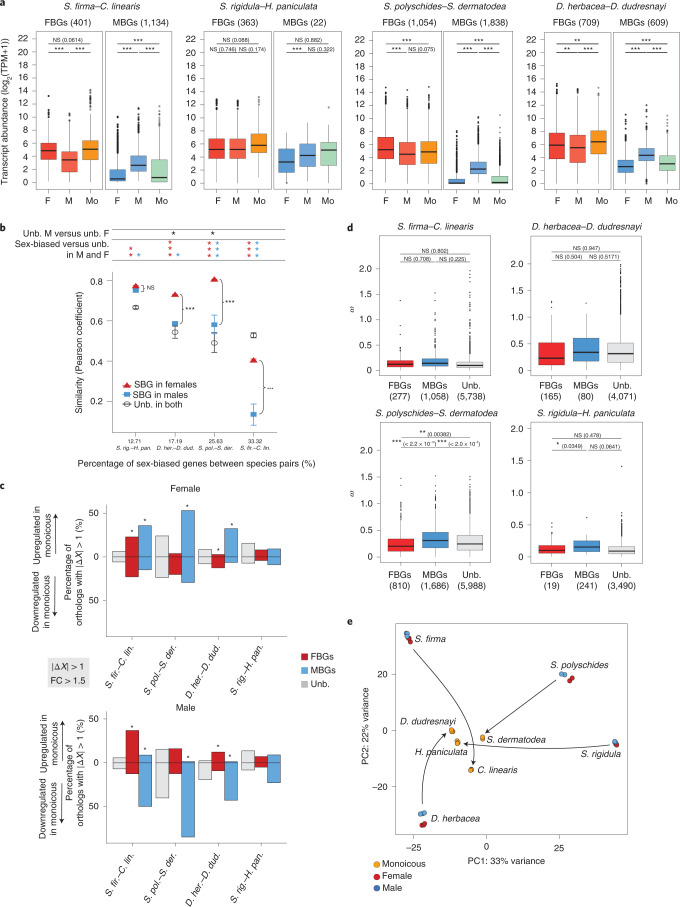
Evolution of sex-biased genes during transitions to monoicy. **a**, Comparison of gene expression levels within species pairs, in log_2_(TPM + 1), using PSO gene sets. Mo, monoicous. The numbers of FBGs and MBGs among PSOs are displayed. Note that only the sex-biased genes with a single-copy ortholog in the corresponding monoicous species are displayed in the plots (in other words, the sex-biased genes represented in the plots are a subset of the sex-biased genes identified within each dioicous species). The boxes represent the interquartile ranges (25th and 75th percentiles) of the data, the lines inside the boxes represent the medians, and the whiskers represent the largest/smallest values within 1.5 times the interquartile range above and below the 75th and 25th percentiles, respectively. The statistical tests are permutation *t*-tests using 100,000 permutations. Paired two-sided *t*-tests were used for comparisons between sexes of the same species (dioicous species). **b**, Comparisons of similarity index values (Pearson coefficients) between expression profiles (in log_2_(TPM + 1)) of PSOs between monoicous and dioicous species pairs. The figure represents male versus female similarity indexes in relation to the monoicous expression profiles. Note that similarity indexes are represented separately for sex-biased genes in females (red) and in males (blue), as well as for unbiased genes averaged across sexes in the dioicous species (black). Pearson coefficients were plotted for each species pair in increasing order of the proportion of sex-biased genes among expressed genes of dioicous species (*x* axis). The asterisks in the top panel represent significant differences between Pearson coefficients, taking into account the correlations between compared gene sets, using the cocor package in R. The red and blue asterisks indicate significant differences between the Pearson coefficients of female (red) or male sex-biased genes (blue) and those of unbiased genes. The black asterisks in the top panel indicate significant differences of Pearson coefficients of unbiased genes between males and females. Significant differences of coefficients between sex-biased genes in females and males are indicated directly on the plot. *0.01 < P < 0.05; **0.001 < P < 0.01; ***P < 0.001. See also Extended Data Fig. 4 and Extended Data Fig. 5. Unb., unbiased; SBG, sex-biased genes. **c**, Fraction of FBGs (red), MBGs (blue) and non-biased genes (grey) with an absolute value of Δ*X* > 1 and FC > 1.5, calculated within species pairs (on PSOs). The percentages are calculated on the total number of orthologs in each category. Downregulated genes in the monoicous species are represented below the *y* = 0 line (Δ*X* < −1); upregulated genes in the monoicous species are represented above the *y* = 0 line (Δ*X* > 1). The asterisks indicate a significant over-representation of FBGs or MBGs with an absolute Δ*X* > 1 compared with the proportion of unbiased genes with Δ*X* > 1, tested using Fisher exact tests. **d**, Sequence divergence, measured as dN/dS (*ω*), between dioicous and monoicous species calculated within species pairs (PSOs). The statistical tests are permutation two-sided *t*-tests using 100,000 permutations, and the *P* values are displayed in parentheses. *0.01 < *P* < 0.05; **0.001 < *P* < 0.01; ***P < 0.001. **e**, Principal component (PC) analysis plot of all the RNA-seq samples, using ASOs. Monoicous species are plotted in orange, female samples in red and male samples in blue.

To study changes in sex-biased gene expression that accompany the transition from dioicy to monoicy, we first identified single-copy orthologous genes for each of the four dioicous–monoicous sister species pairs (pairwise single-copy orthologs (PSOs); Fig. 4a). We were able to infer between 6,109 and 11,953 PSOs for each of the four pairs of species (Fig. 4a and Supplementary Tables 7–11). PSOs were classified as being sex-biased or unbiased by comparing male and female expression in each dioicous species (false discovery rate < 0.05, FC > 2). We then examined the patterns of expression of MB, FB and unbiased PSOs in dioicous males and females and in the corresponding monoicous species.

**Extended Data Fig. 4:**
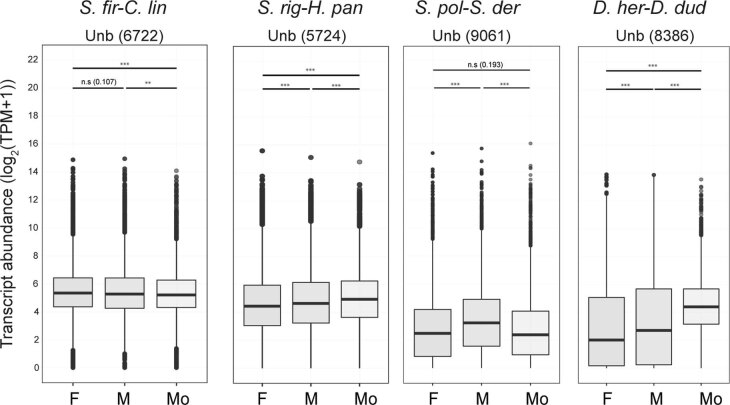
Comparison of unbiased genes expression levels within species pairs, in log2(TPM+1), using PSO gene sets. F: females. M: males. Mo: monoicous. Boxes represent the interquartile range (25th and 75th percentiles) of the data, the line inside the box represents the median, whiskers represent the largest/smallest value within 1.5 times interquartile range above and below the 75^th^ and 25^th^ percentile, respectively. Statistical tests are permutationtwo-sided *t*-tests using 100,000 permutations. Paired *t*-test were used for comparisons between sexes of the same species (dioicous species). ** 0.001 < P < 0.01; *** P < 0.001. Number of analysed genes are presented inside brackets.

In three of the four species pairs, the levels of expression of sex-biased genes in the monoicous species were similar to the values measured for orthologs in females of the corresponding dioicous species (Fig. 4a and Extended Data Fig. 4). In these three pairs, MBGs were downregulated in the monoicous species compared with males, and they displayed similar expression levels to MBGs in females of the dioicous species, suggesting that de-masculinization of gene expression of the monoicous species counterpart occurred frequently. FBGs were expressed at similar mean levels in *S. firma* females compared with the corresponding monoicous species *Chordaria* *linearis*. In the *S. polyschides–Saccorhiza* *dermatodea* pair of species, FBGs had a similar pattern in males and monoicous individuals. Both FBGs and MBGs in *D. herbacea* showed significantly different mean expression levels compared with *Desmarestia* *dudresnayi*. In the *S. rigidula–Halopteris* *paniculata* species pair, no significant difference was detected between the expression of sex-biased and unbiased genes between the two species. Note, however, that the results for *S. rigidula–H. paniculata* were more difficult to interpret, as the low number of sex-biased genes among the PSOs precluded robust statistical analysis.

**Extended Data Fig. 5:**
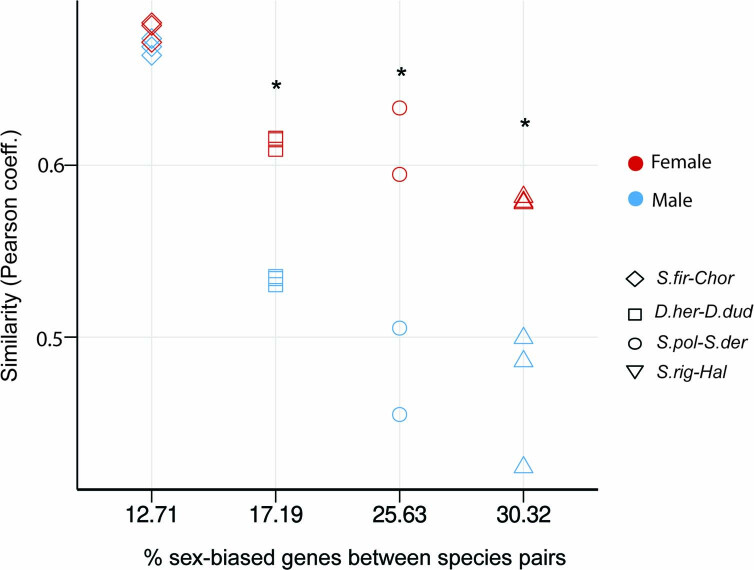
Comparisons of similarity index values (Pearson coefficients) between expression profiles (in log_2_(TPM + 1)) of all orthologous genes of dioicous and monoicous species within species pairs (PSO), per sex. Similarity index are represented separately for each female replicate sample (red) and male replicate sample (blue). Asterisks represent significant differences between Pearson coefficients, taking into account the correlations between compared gene sets, tested using a two-sided significance test the cocor package in R.

**Extended Data Fig. 6:**
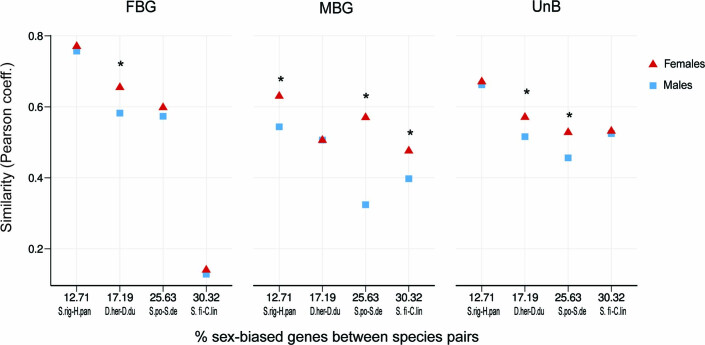
Comparisons of similarity index values (Pearson coefficients) between expression profiles (in log_2_(TPM+1)) of orthologous genes between dioicous and monoicous species within species pairs (PSO), per category of gene. Similarity index are represented separately for mean female (red) and mean male (blue) expression profiles and for three categories of orthologous genes: male-biased genes in the dioicous species (MBG), female-biased genes in the dioicous species (FBG) and unbiased genes (UnB). Asterisks represent significant differences between Pearson coefficients, taking into account the correlations between compared gene sets, tested using a two-sided significance test with the cocor package in R.

We next investigated the gene expression profiles of monoicous species to test whether their transcriptional patterns resemble those of their male or female dioicous counterparts. We computed the Pearson product-moment coefficient of regressions of gene expression profiles (in log_2_(TPM + 1)) of males or females compared with that of the monoicous species within each species pair. We compared Pearson correlation coefficients for both sex-biased genes and unbiased genes in males or females, considering sex-biased genes in males and females as independent groups. We also compared the correlations of expression profiles with the orthologs of sex-biased and unbiased genes in the monoicous species, separately for males and females. We considered these groups of sex-biased versus unbiased genes being expressed within the same individuals as dependent groups in the cocor package^40^. Altogether, these analyses indicated that, except for the *S. rigidula–H. paniculata* species pair, the gene expression profiles of the monoicous species were significantly more similar to those of females than they were to male profiles (Fig. 4b and Extended Data Fig. 5). Moreover, the close association between female and monoicous expression profiles was observed for both sex-biased and unbiased genes specifically in the *Saccorhiza* and *Desmarestia* species pairs (Fig. 4b, black asterisks at the top; see also Extended Data Fig. 6).

Interestingly, except for the Ectocarpales species pair (*S. firma*–*C. linearis*), sex-biased gene expression profiles diverged significantly less from the monoicous species than did those of the unbiased genes (Fig. 4b and Extended Data Fig. 5). Overall, the expression profile similarity observed between females and monoicous individuals was mainly driven by expression patterns of MBGs, except in the *Desmarestia* species pair (Fig. 4a and Extended Data Fig. 5). We also noted that the highest similarity indexes for within-species pairs were found for the species with the lowest level of sex-biased gene expression (*S. rigidula*), and the lowest similarity was observed for the species with the highest level of sex-biased gene expression (*S. firma*) (Fig. 4b). The relatively high proportion of sex-specific genes present in *S. firma* (Fig. 2a) is unlikely to be the cause of the observed low similarity, because only 29 (0.49%) of the sex-specific genes are among the PSOs used for the Pearson similarity analysis.

Taken together, the above observations suggest that the gene expression profiles of monoicous species tend to be more closely related to those of the females of the related dioicous species, and this similarity seems to be driven by sex-biased genes, particularly MBGs. The tendency to reproduce the female transcriptome in the monoicous species was repeatable in independent transitions to co-sexuality.

### Is selection involved in expression changes during transitions to monoicy?

To examine whether changes in gene expression during transitions to co-sexuality were the result of selective or neutral forces, we computed the degree of directional selection using Δ*X*. This parameter evaluates the divergence in expression level in relation to the variation in expression level seen across replicates^11,36,41^. We computed Δ*X* of the PSO sets separately for each pair of species and reported the proportions of orthologs with an absolute Δ*X* > 1—that is, orthologs whose expression shift is attributable to directional selection (Supplementary Table 11 and Fig. 4c). Depending on the species pair, between 10.8% and 40.1% of unbiased genes exhibited expression shifts attributable to selection (|Δ*X*| > 1) (Supplementary Table 12). We then asked whether MBGs and FBGs were preferentially concerned by adaptive expression shifts during transitions to monoicy compared with unbiased genes. Figure 4c illustrates the proportion of orthologs with sex bias displaying |Δ*X*| > 1 (in other words, under putative directional selection) and how sex-biased genes are more likely to display |Δ*X*| > 1 than are the unbiased orthologs. Fisher’s exact tests (the asterisks in Fig. 4c) showed that for three of the four species pairs, MBGs were indeed more likely to display |Δ*X*| > 1 than unbiased genes (Fig. 4c). This was also the case for FBGs in the *S. polyschides–S. dermatodea* pair (Fisher exact tests, *P* < 2.2 × 10^−16^ in both sexes) and the *D. herbacea–D. dudresnayi* pair (Fisher exact tests, *P* = 3.9 × 10^−3^ and *P* = 1.8 × 10^−5^ in females and males, respectively). In *S. polyschides–S. dermatodea* and *S. rigidula–H. paniculata*, FBGs showed lower levels of adaptive evolution of expression compared with unbiased genes (Supplementary Table 11 and Fig. 4c). Taken together, our observations indicate that MBGs preferentially exhibit a shift in expression during the transition to monoicy that may be explained by directional selection.

We also assessed whether the evolution of gene expression during the transition to monoicy has been driven by DNA sequence evolution, by using measures of sequence divergence (dN/dS). We computed dN/dS for MBGs, FBGs and unbiased genes for each of the dioicous–monoicous species pairs. For all four pairs, MBGs consistently exhibited higher evolutionary rates than FBGs and unbiased genes, although this difference was significant only for the *S. polyschides–S. dermatodea* pair (Fig. 4d and Supplementary Table 13). As this is the ‘youngest’ species pair (Fig. 1), it seems that the level of sequence divergence during the transition to monoicy is not associated with the age of transition. Taken together, our observations indicate that shifts from dioicy to monoicy involved modifications to transcriptional patterns (expression divergence) mostly at MBGs that were probably driven by selection but also by coding sequence evolution.

### Convergent gene expression changes during transitions to monoicy

To assess the extent to which gene expression changes occurring during the transition to monoicy were shared across the four species pairs, we focused on the single-copy orthologs across the eight species, herein termed ‘all single-copy orthologs’ (ASOs). We found a total of 1,708 ASOs (following the same approach as for DSOs; Methods).

**Extended Data Fig. 7:**
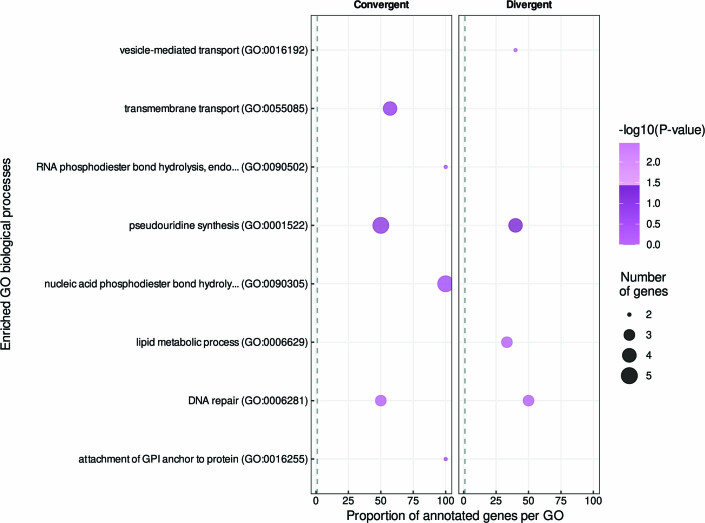
GO term analysis. Functional analysis (enriched GO terms for biological processes, tested with Fisher’s exact Test, P_adj_ < 0.01) of genes that are convergently or non-convergently expressed in dioicous versus monoicous species.

Among the 1,708 ASOs, 718 were sex-biased in at least one dioicous species (Supplementary Tables 14 and 15). Sex-biased genes were not over-represented among ASOs (Fisher exact test, *P* = 0.097). Sixty-one per cent of the ASOs (1,043 of 1,708) exhibited a conserved pattern of expression across all monoicous species compared with the dioicous species. This proportion was significantly different from what was expected by chance (permutation tests, *P* = 0.0255, 10,000 permutations), suggesting convergent gene expression changes during transitions to monoicy across all studied pairs of species. Decomposition of variance components for the 1,708 ASOs detected a clear pattern of grouping of monoicous species, further illustrating the extensive convergence of gene expression during the transition from dioicy towards monoicy (Fig. 4e). Functional analysis of genes that are convergently expressed during the transition to monoicy highlighted terms such as nucleic acid metabolic processes and transmembrane transport (Extended Data Fig. 7).

About half (527) of the 1,043 genes that were consistently differentially expressed in monoicous versus dioicous species had a |Δ*X*| > 1, which is significantly more in proportion than among the rest of the ASOs (290 genes with |Δ*X*| > 1 among 665 ASOs, Fisher exact test, *P* = 0.00543). This observation indicates that convergent gene expression changes may be associated with directional selection during the switch to monoicy.

**Extended Data Fig. 8:**
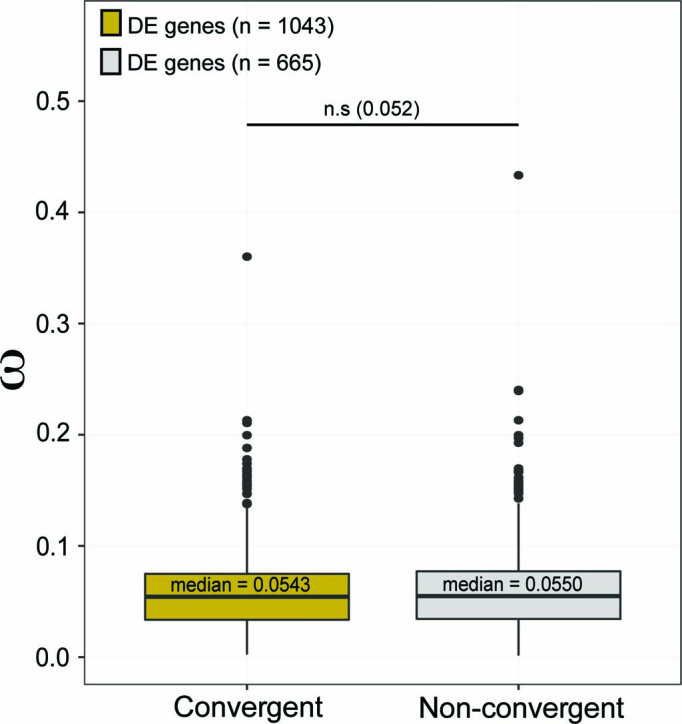
Sequence divergence, measured as dN/dS (ω), between convergent and non-convergent genes across all species pairs (ASO). Boxes represent the interquartile range (25th and 75th percentiles) of the data, the line inside the box represents the median, whiskers represent the largest/smallest value within 1.5 times interquartile range above and below the 75^th^ and 25^th^ percentile, respectively.

We next tested whether sexual selection potentially occurring in males and females of dioicous species would be relaxed in monoicous individuals. This would translate to a reduction of purifying selection resulting in increased sequence divergence (increased dN/dS). Convergent genes (that is, genes exhibiting a convergent pattern of gene expression in monoicous species) tended to exhibit faster divergence rates than non-convergent genes, although the difference was not significant (permutation *t*-test, *P* = 0.0566; Extended Data Fig. 8). Notably, MBGs (but not FBGs) showed significantly higher dN/dS values than unbiased genes (Supplementary Table 16).

A likelihood ratio test of branch models (after Benjamini–Hochberg correction for multiple testing) identified 689 orthologs under positive selection on monoicous branches, 404 of which exhibited convergent gene expression changes. Orthologs under positive selection were over-represented among genes with convergent gene expression (Fisher exact test, *P* = 0.025). Taken together, these observations suggest that directional selection plays a role in driving changes in expression patterns during transitions to co-sexuality.

## Discussion

### Sexual dimorphism and sex-biased gene expression are uncoupled

Morphological and physiological differences between males and females are ultimately due to divergences between sex chromosomes in species with genetic sex determination^27^, but the majority of morphological sexual dimorphism is thought to be associated with autosomal sex-biased gene expression^9–11^. It would thus be expected that species showing more prominent differences in morphology between males and females would also be characterized by high levels of sex-biased gene expression, as has been shown to be the case in birds^37^. Our study, in contrast, revealed no correlation between the level of sex-biased gene expression and the degree of phenotypic sexual dimorphism in the brown algae studied here. The link between gene expression evolution and sexual selection is therefore uncertain for these organisms, and sexual selection is likely not to be the main driver of sex-biased gene expression evolution. This observation may reflect a lower degree of sexual selection in brown algae than in animals. Brown algae have relatively low levels of sexual dimorphism^15,29^ and are broadcast spawners, so the opportunities for mate choice and/or mating competition are mainly constrained to interactions involving male and female gametes^42^. Consistent with the idea that gamete sexual selection may occur, it has been shown recently that in the absence of males, female gametes of brown alga populations lose their sexual morphological characteristics—for example, female gametes produce lower levels of pheromone and engage in parthenogenesis more rapidly^43^. Notably, sex-biased genes found in male and female gametophytes of the model brown alga *Ectocarpus* show more rapid rates of divergence across species (measured as dN/dS) compared with unbiased genes, and their accelerated evolution has been at least partly attributed to positive selection^15^. These observations suggest that sexual selection plays a role in the evolution of sex-biased genes in brown algae but may not be the only driver of sex-biased gene expression in this group of organisms.

### Sex-biased genes exhibit functional convergence

Although dioicy is predicted to be the ancestral sexual system in brown algae^32^, our results clearly indicate that sex bias in the expression of individual genes is neither ancestral nor convergent. We found a very limited level of shared (ancestral) sex-biased gene expression across the studied brown algal species, and instead our data are consistent with lineage-specific recruitment of sex-biased genes. Our observations therefore emphasize a substantial turnover of sex-biased expression among brown algal genes.

Interestingly, our study suggests that sex-biased expression may have emerged on genes that were experiencing lower selective constraints on their expression levels, possibly due to lower pleiotropy, in addition to the potential effect of sex-specific selection occurring after the evolution of separate sexes. A similar situation has been described recently in plants^36^ and animals^39^.

Although the dioicous brown algal species studied here shared very few sex-biased genes, we found some level of convergence in terms of sex-biased gene function, at least for a subset of the sex-biased genes. These include biological functions that were previously found to be enriched in *Ectocarpus* gametophytes^15,44^, further underscoring the conservation of sex-biased gene function and supporting primary sexual dimorphic roles. These functions may be associated with sex-specific biological processes. For example, enrichment in oxidation–reduction functions may relate to the more conspicuous growth of female gametophytes, producing larger gametes that secrete a sperm-attracting pheromone, whereas cilia and ion transport functions are probably associated with the production of fast-swimming, bi-flagellated sperm by male gametophytes. Considering that brown algae share an ancestral sex chromosome, and that genes within the non-recombining sex-determining region play a role in sex^45^, one possibility is that sexual characteristics in these UV systems mainly involve genes in the sex-determining region together with a relatively limited number of autosomal genes involved in primary sexual dimorphisms. In other words, differences between sexes arise mainly from the different physiological processes directly linked to the production of male or female gametes rather than extensive sexual selection, sexual specialization and/or sexual antagonism (that is, secondary sexual dimorphism)^12^.

### Fate of sex-biased gene expression during transitions to monoicy

Our sampling of species distributed across the brown algae phylogeny, associating pairs of related dioicous and monoicous species, allowed us to trace the fate of sex-biased gene expression during independent events of transition from dioicy to monoicy. Except in one species pair, sex-biased genes exhibited adaptive expression shifts during the transition to monoicy. MBGs, specifically, were the main drivers of gene expression changes during the transition to monoicy, while unbiased genes exhibited limited changes in expression patterns with the switch in sexual system. In the model brown alga *Ectocarpus*, RNA-seq analysis of multiple tissues and life cycle stages indicated that sex-biased genes have restricted patterns of expression, which is a proxy for limited pleiotropy^15^. Pleiotropy is known to restrict gene evolution, imposing stricter functional constraints on pleiotropic genes^39,46^. The reduced pleiotropy of sex-biased genes compared with unbiased genes may increase their propensity to adaptively shift towards their evolved optimal expression profile during evolutionary transitions, in this case the transition to monoicy^10,39,47^.

Sex-biased genes in dioicous brown algae such as *Ectocarpus* spp. typically display higher evolutionary rates than unbiased genes due to either directional selection or relaxed purifying selection^15^. With the transition to monoicy, increased relaxation of sex-specific purifying selection acting on sex-biased genes may be expected, leading to increased rates of sequence evolution. Accordingly, MBGs for all species pairs presented faster evolutionary rates (although not significant for all species) during the switch to monoicy, compared with FBGs or unbiased genes. This observation points to a shared process of sexually antagonistic selection within dioicous species, especially in males, that allowed for faster evolutionary rates of MBGs when relaxed during the transition from dioicy to monoicy.

### Convergent changes during the breakdown of dioicy

Convergent evolution, where a similar trait evolves in different lineages, provides an opportunity to study the repeatability of evolution. In the brown algae, co-sexuality has repeatedly emerged from unisexual ancestors^32^. We found that more than half (61%) of the orthologs across the four pairs of species displayed similar expression shifts concomitant with the transition to monoicy, indicating that common, independently acquired mechanisms are associated with co-sexuality. Remarkably, a substantial number of these convergent genes (38.7%) were under positive selection, underlying the idea that convergent changes associated with the shift of sexual system may be driven by comparable evolutionary pressures across these distant species. Monoicous gametophytes were more closely related to females of the corresponding dioicous species counterpart, suggesting (as proposed in volvocine algae^48^) that monoicy may have arisen from ancestral females.

In our study, the expression profiles of gametophytes of all four monoicous species resembled those of the female gametophytes of their dioicous counterparts. Moreover, sex-biased genes tended to maintain the levels of expression they had in dioicous species, suggesting that they retained their ancestral functions in the context of derived monoicy. When their expression shifted, sex-biased genes (especially MBGs) showed signs of selection acting on their expression levels to a greater extent than it acted on unbiased genes. Together, our results demonstrate that common mechanisms underlie the transition to monoicy across distant brown algal lineages and suggest that independent events of loss of dioicy may have involved the acquisition of genes related to male development by a female gametophyte. The work presented here therefore establishes a framework for understanding at the genomic level how co-sexual systems arise from ancestral haploid UV sexual systems in the brown algae.

## Methods

### Sample preparation, RNA extractions and sequencing

The algal strains used and the sequencing statistics and BioProject accession numbers are listed in Supplementary Table 1. Gametophytes of all eight species were cultured at 13 °C in autoclaved natural sea water supplemented with half-strength Provasoli solution (PES^49^) with a light:dark cycle of 12 h:12 h (20 µmol photons per m^2^ per s) using daylight-type fluorescent tubes^50^. All manipulations were performed under a laminar flow hood in sterile conditions. Immature gametophytes (that is, without sex-specific reproductive structures, oogonia or antheridia) of each strain were frozen in liquid nitrogen and kept at −80 °C until RNA extraction.

RNA from male and female pools was extracted from triplicate samples (each containing at least 800 individual gametophytes, except for *S. polyschides* and *S. dermatodea*, where two replicates were used) using a Qiagen RNA extraction Plant Mini kit. RNA quality and quantity were assessed using an Agilent 2100 bioanalyser, associated with an RNA 6000 Nano kit. For each replicate, the RNA was quantified and cDNA was synthesized using an oligo-dT primer. The cDNA was fragmented, cloned and sequenced by Fasteris using Illumina HiSeq 2000 for the *Saccorhiza* and *Desmarestia* species, by Genome Quebec using a Nextgen6000 for the *Halopteris* and *Chordaria* species, and by Genoscope using Illumina HiSeq 4000 for the *Sphacelaria* and *Sphaerotrichia* species (see Supplementary Table 1 for the details).

### Transcriptome assemblies and gene set predictions

Predicted gene sets were constructed for each species on the basis of genome and transcriptome assemblies. To filter out potential contamination, first-round assembled contigs were blasted against the NCBI non-redundant protein database using diamond v.0.9.21 (ref. ^51^), and reads that mapped on contigs with non-eukaryotic taxa were removed using blobtools v.1.0.1 (ref. ^52^). De novo transcriptomes were assembled using Trinity (*S. polyschides*, *S. dermatodea*, *D. dudresnayi, D. herbacea* female, *H. paniculata* and *S. rigidula*) or rnaSPADES v.3.12.0 (*C. linearis* and *S. firma*) with a *k*-mer size of 55.

All genomes were soft-masked using Repeatmasker v.4.0.9 after building a de novo transposable elements and repeats database with RepeatModeler v.1.0.8 (ref. ^53^). BRAKER2^54^ and PASA (for *D. herbacea*^55^), using input predicted protein from the reference species *Ectocarpus* sp. (EctsiV2_prot_LATEST.tfa^56^), were used to predict gene sets used for all downstream analyses.

The final assemblies are available in NCBI (BioProject accession number PRJNA733856). Transcriptome completeness was assessed using the BUSCO v.3 eukaryote gene set as a reference (Odb9). Transcripts that had DNA data support for only one sex (potentially sex-linked) were tested with PCR using at least four males and four females per species and were removed from the sex-biased gene analysis. The PCR primers are detailed in Supplementary Table 17.

### Expression quantification and inference of sex-biased genes

RNA-seq reads adaptors were trimmed using trimmomatic v.0.38 (ref. ^57^), which was also used for read-quality filtering: reads were removed if the leading or trailing base had a Phred score <3 or if the sliding-window Phred score, averaged over four bases, was <15. Reads shorter than 36 bases were discarded (as well as pairs of reads if one of the pair was <36 bases long). Trimmomatic-processed RNA-seq reads from each library were used to quantify gene expression with kallisto v.0.44.0^58^ using 31-base-pair-long *k*-mers and predicted transcripts of each species. The RNA-seq libraries were composed of stranded (fr-stranded or rf-stranded option) single-end reads (single option) or paired-end reads (Supplementary Table 1). A gene was considered expressed in a given species and/or sex when at least one library displayed an expression level (in TPM) above the fifth percentile of the TPM distribution across all genes and libraries within a species and sex. Following ref. ^59^, transcript abundances were then summed within genes and multiplied by the total library size, using the tximport package^35^ to obtain the expression level for each gene in TPM.

Estimates of sex-biased gene expression in dioicous species were obtained using read count matrices as input for the DESeq2 package^35^ in R v.3.6.3. *P* values were corrected for multiple testing using Benjamini and Hochberg’s algorithm in DESeq2, applying an adjusted *P*-value cut-off of 0.05 for differential expression analysis. In addition, only genes with a minimum of 2FC expression level between sexes were retained as sex-biased.

### Quantification of phenotypic sexual dimorphism

Individual gametophytes from each strain were isolated in sea water and observed using an inverted transmitted light microscope DMi8 (Leica) with LAS X software. Between 269 and 556 cells (348 cells on average per sex and per species) across five different gametophytes per species were individually measured using Fidji^60^. We used *t*-tests to compare cell length between groups. The difference in mean cell length between sexes of dioicous species was computed and used as a proxy for phenotypic sexual dimorphism. To investigate the relationship between phenotypic sexual dimorphism and extent of sex-biased expression, phenotypic dimorphism was regressed against the fraction of sex-biased genes in R, corrected for phylogeny using the phylogenetic generalized least squares method as implemented in the nlme R package^61^.

### Orthology and evolutionary rates within species pairs

We inferred PSOs within the four species pairs using Orthofinder with the default parameters^62^. We used kallisto v.0.44.0 to quantify the expression levels for PSOs within species pairs.

To infer the potential role of selection in expression changes between dioicous and monoicous species, we computed Δ*X*. To summarize, we calculated Δ*X* = *d*/*r* with *d* and *r* given by:

$$d = ({\mathrm{Mean}}\,X_{{\mathrm{dioicous}}} - {\mathrm{Mean}}\,X_{{\mathrm{monoicous}}})/{\mathrm{Mean}}\,X_{{\mathrm{dioicous}}}$$ and

$$r = [ ({X_{{\mathrm{dioicous}}}} )^{{\mathrm{high}}} - ( {X_{{\mathrm{dioicous}}}} )^{{\mathrm{low}}}]/{\mathrm{Mean}}\,X_{{\mathrm{dioicous}}}$$

where *X* is the expression level measured in TPM, and ‘high’ and ‘low’ represent the maximum and minimum values. Δ*X* was computed separately for females and males of the dioicous species and for MBGs, FBGs and unbiased genes. orthologs with |Δ*X*| > 1 and a minimum expression FC between sister species of 1.5 were considered to have had a significant evolutionary expression shift. Fisher exact tests were computed to detect whether FBGs and MBGs were more likely to show an absolute value of Δ*X* > 1 than unbiased genes.

Orthologous proteins between species pairs were aligned with MAFFT v.7.453 (ref. ^63^), and the alignments were curated with Gblocks v.0.91b^64^ and back-translated to nucleotides using translatorX^65^. We used these nucleotide alignments as input for phylogenetic analysis by maximum likelihood (PAML4, CodeML^66^) to infer pairwise dN/dS (*ω*) with the F3x4 model of codon frequencies. We retained orthologs with 0 < dS < 2 as valid for further analysis. We compared species’ and sexes’ evolutionary rates separately for FBGs, MBGs and unbiased genes, using permutation *t*-tests in R with 100,000 permutations.

### Evolution of sex-biased gene expression

We inferred a single orthologous gene set for the four dioicous species (DSOs) using Orthofinder with the default parameters. Following the methods used in ref. ^67^, we included in the DSOs the OGs with genes that were 1:1:1:0, probably due to situations in which a single-copy ancestral gene was lost in a single species. To further account for gene prediction errors, we also included OGs with a single species presenting two genes that aligned on more than 60% of their length as duplicate genes. In the latter case, the longest duplicated sequence was retained for further analysis.

A well-resolved phylogeny of the Phaeophyceae was used as the reference gene tree^34^ to infer where sex-biased gene expression evolved along the phylogenetic tree. We coded DSOs as either MB, FB or unbiased for each species and used the ape package^68^ in R to reconstruct the discrete ancestral state via maximum likelihood. Proportions of ancestral genes in each category were plotted as pie charts on tree nodes, and gain and loss of bias were reported on each branch. We further tested the significance of overlap between sex-biased genes identified within dioicous species with exact multi-set intersection tests implemented in the SuperExactTest package v.1.0.7 in R^69^.

We computed absolute standardized PICs among dioicous species, using the ape package in R. Mean PICs were compared using Mann–Whitney rank tests between unbiased genes and sex-biased genes, with their expression measured in species in which they were not sex-biased.

We inferred expression profile similarity indexes between monoicous species and males and females of dioicous species within pairs as the Pearson correlation coefficient of PSO expression levels in log_2_(TPM + 1). This analysis was performed for all expressed genes and separately for MB, FB and unbiased genes. We compared Pearson coefficients of regression within each species pair using the cocor package^40^, considering the gene expression profiles of males and females as independent gene sets. We also compared sex-biased genes with unbiased genes within sexes, considering these gene sets as dependent. We report the *P* value based on Fisher’s *z* or, when possible, Silver, Hittner and May’s modification of Dunn and Clark’s *z*. Pearson’s coefficients were plotted for each species pair.

### Convergent expression changes

Convergent changes associated with transitions to monoicy were investigated on single-copy orthologs inferred across the eight studied species (termed ASOs) following the same methods as those used for the DSOs. Using this dataset, we quantified gene expression with kallisto as described above, and DESeq2 was used to infer orthologs significantly affected by sexual system but not species pair (lfcShrink with the ‘ashr’ method, sexual system contrast^70^). The significance of the number of convergent expression changes was tested with permutation tests (100,000 permutations). We used the ComplexHeatmap package in R to visualize gene expression for each replicate. OGs with inconsistent sex bias across different species (*n* = 139) were removed from the dN/dS analysis of convergent gene evolution.

Intersects between genes across PSOs, DSOs and ASOs were represented using the UpSetR package v.1.4.0 (ref. ^71^).

### ASO evolutionary rates

Following the same process described for PSOs, we aligned and studied molecular sequence divergence for ASOs using CodeML. We used a ‘two-ratio’ branch model (model = 2, Nssites = 0) to specifically study divergence on monoicous branches (foreground branches). We compared *ω* values separately between sex-biased (MB and FB) and unbiased genes with permutation *t*-tests (10,000 permutations). We also ran two branch-site models in PAML to detect positive selection in foreground branches (model = 2, Nssite=2, *ω* = 1 fixed (Null) or allowed to vary). Likelihood ratio tests were used to compare the model of selection with the null model to detect orthologs with sites under positive selection in the monoicous branches. Likelihood ratio test *P* values were corrected for multiple testing using Benjamini and Hochberg’s algorithm^72^.

### Functional annotation analysis

Predicted genes and OGs were blasted against the NCBI non-redundant protein database with blast (v.2.9.0). Functional annotation was performed using Blast2GO^38^, as well as the InterProScan prediction of putative conserved protein domains^73^. Gene set enrichment analysis was carried out separately for each gene set using Fisher’s exact test implemented in the TopGO package, with the weight01 algorithm^74^. Values were corrected for multiple testing using the Benjamini–Hochberg method to control the false discovery rate. We investigated enrichment in terms of biological process ontology and reported significant GO terms with *P* < 0.01. All statistical analyses were performed in R v.3.6.3, and the plots were produced with ggplot2 in R (https://ggplot2.tidyverse.org/).

### Reporting Summary

Further information on research design is available in the Nature Research Reporting Summary linked to this article.

## Supplementary information

Reporting Summary

Peer Review Information

Supplementary Tables Excel file with Supplementary Tables 1–18.

## Data Availability

The raw reads have been deposited in the SRA. The BioProject accession number is PRJNA733856. The accession codes are given in Supplementary Table 18.
