# Supplementary material for: Selection drives convergent gene expression changes during transitions to co-sexuality in haploid sexual systems
Source: Nat Ecol Evol. 2022 Mar 21;6(5):579–89. doi: 10.1038/s41559-022-01692-4 (PMC9085613; doi:10.1038/s41559-022-01692-4)
Supplement: Supplementary file 2 — Peer Review Information [file 41559_2022_1692_MOESM2_ESM.pdf]

natureresearch

Peer Review Information

Journal: Nature Ecology & Evolution
Manuscript Title: SELECTION DRIVES CONVERGENT GENE EXPRESSION CHANGES DURING TRANSITIONS TO CO-SEXUALITY IN HAPLOID SEXUAL SYSTEMS
Corresponding author name(s): Susana M. Coelho

Editorial Notes:

Reviewer Comments & Decisions:

Decision Letter, initial version:

29th July 2021

*Please ensure you delete the link to your author homepage in this e-mail if you wish to forward it to your co-authors.

Dear Dr Coelho,

Your manuscript entitled "SELECTION DRIVES THE EVOLUTION OF CONVERGENT GENE EXPRESSION CHANGES DURING TRANSITIONS TO CO-SEXUALITY IN HAPLOID SEXUAL SYSTEMS" has now been seen by three reviewers, whose comments are attached below. The reviewers have raised a number of concerns which will need to be addressed before we can offer publication in Nature Ecology & Evolution. We will therefore need to see your responses to the criticisms raised, along with a revised manuscript, before we can reach a final decision regarding publication.

We therefore invite you to revise your manuscript taking into account all reviewer and editor comments. Please highlight all changes in the manuscript text file.

We are committed to providing a fair and constructive peer-review process. Do not hesitate to contact us if there are specific requests from the reviewers that you believe are technically impossible or unlikely to yield a meaningful outcome.

When revising your manuscript:

* Include a “Response to reviewers” document detailing, point-by-point, how you addressed each

reviewer comment. If no action was taken to address a point, you must provide a compelling argument. This response will be sent back to the reviewers along with the revised manuscript.

* If you have not done so already please begin to revise your manuscript so that it conforms to our Article format instructions at http://www.nature.com/natecolevol/info/final-submission. Refer also to any guidelines provided in this letter.

* Include a revised version of any required reporting checklist. It will be available to referees (and, potentially, statisticians) to aid in their evaluation if the manuscript goes back for peer review. A revised checklist is essential for re-review of the paper.

Please use the link below to submit your revised manuscript and related files:

[REDACTED]

Note: This URL links to your confidential home page and associated information about manuscripts you may have submitted, or that you are reviewing for us. If you wish to forward this email to co-authors, please delete the link to your homepage.

We hope to receive your revised manuscript within four to eight weeks. If you cannot send it within this time, please let us know. We will be happy to consider your revision so long as nothing similar has been accepted for publication at Nature Ecology & Evolution or published elsewhere.

Nature Ecology & Evolution is committed to improving transparency in authorship. As part of our efforts in this direction, we are now requesting that all authors identified as 'corresponding author' on published papers create and link their Open Researcher and Contributor Identifier (ORCID) with their account on the Manuscript Tracking System (MTS), prior to acceptance. ORCID helps the scientific community achieve unambiguous attribution of all scholarly contributions. You can create and link your ORCID from the home page of the MTS by clicking on 'Modify my Springer Nature account'. For more information please visit please visit www.springernature.com/orcid.

Please do not hesitate to contact me if you have any questions or would like to discuss these revisions further.

We look forward to seeing the revised manuscript and thank you for the opportunity to review your work.

[REDACTED]

Reviewer expertise:

Reviewer #1: evolution of sexual systems and sex chromosomes

Reviewer #2: evolution of sexual dimorphism, sex-biased gene expression

Reviewer #3: evolution of sexual systems and sex chromosomes

Reviewers' comments:

Reviewer #1 (Remarks to the Author):

Cossard et al provide a detailed analysis of the evolution of gene expression levels in eight species of brown algae sampling 4 independent transitions from separated sexes (dioicy, the ancestral state) to cosexuality. Their main result, I find, is that the evolution of cosexuality is accompanied by a convergence in gene expression among the four independently evolved cosexual species. Figure 4E remarkably illustrates this finding. Other noteworthy findings are that (i) the amount of sex-biased genes in dioicous species is not correlated to the level of sexual dimorphism, which confirms results obtained on the plant genus Leucadendron and add up to the potential distinction between animals and plants+algae, (ii) cosexuals species expression resembles female expression from dioicous species, suggesting that maybe cosexual species have evolved from females, (iii) sex-biased gene expression has a high turnover.
The manuscript is well written. I however have a few concerns about clarity (mainly regarding Figures) and one additional analysis that I would like to suggest.

Are monoicous species UU? If this is unknown the authors could at least discuss this question. This would give some more perspective to the expression resemblance between females and cosexuals.

Throughout the manuscript, sex-biased expression is interpreted in terms of adaptation (for example Lines 241-243). However, Scharmann et al 2021 Biorxiv very convincingly show that sex-biased expression could be the result of random expression evolution under low selective pressures for non pleiotropic genes. I suggest the authors do a similar analysis to Scharmann et al 2021 Biorxiv and look at the rate of expression evolution of genes orthologous to sex biased genes in species where the genes are not sex biased.

Were Figure 2E correlations corrected for phylogeny using PGLS?

Lines 169-171: I don't find the result of convergence in sex-bias expression striking in dioicous species because for example photosynthesis and oxidation/reduction processes appear in only 2 of the 4 species in Figure 3C. I did not check all terms but maybe the authors could find a more convincing statistical way to say that the terms are non randomly found in common in the different species? What is the chance given the number of GO terms and genes that one term is found in common in two species?
Same Line 303: I wouldn't speak about remarkable convergence because it seems to me that just a few sex-biased genes involved in a similar function are enough for the term to appear in multiple species. So this does not mean that all sex-biased genes are involved in convergent functions, but rather that at least a few of them are.

I don't understand FIgure 3A, what intersect is it representing?

I would like to see Figure 4A for unbiased genes, just to check for any potential expression normalization issues.

In Figure 4A, FBG in S. rigidula don't look very female biased. Even the ns symbol says there is no

difference between F and M expression for these genes. Same for MBG in these species. There seems to be an issue there. If these genes were identified as sex-biased with two-fold change why do they have ns expression difference on Figure 4A? Are statistical tests paired in Figure 4A across genes? The statistical test should be paired because gene expression in females is dependent on gene expression in males.

Lines 181-192 When looking at significance levels in Figure 4A the conclusion is that monoicous expression is often significantly DIFFERENT from female expression. Looking at bars it is true that monoicous expression seems closer to female expression than to male expression but this is very descriptive rather than quantitative. I think in this paragraph the authors should not conclude that monoicous expression is closer to female, this conclusion rather comes from Figure 4B.
Line 187 the expression levels are significantly different between herbacea females and the corresponding monoicous species, please rephrase.

Figure 4B is difficult to interpret. I would favor a less condensed representation because I don't understand where is the pearson coefficient between dioicous females and monoicous and where is the pearson coefficient between dioicous male and monoicous. All pearson coefficients should be shown separately for males and females and also for different gene sets (MBG, FBG and unbiased genes) The Figure only shows sex-biased genes in females and males but these gene sets have different male and female expression. I might have misunderstood completely Figure 4B, in which case a clarification would be welcome.

Figure 4C has two panels, one for male and one for female. Was delta x computed separately for dioicous male and female expression? This was very unclear from the methods and main text and should be clarified.

Generally speaking, the Figures would benefit from additional color legends directly on the graphs and more detailed axis names because many graphs cannot be understood without reading the legend.

Line 209-211: Could this be due to a normalization issue? When many genes have sex-biased expression they affect other genes expression because they take up a high proportion of transcripts per million, making pearson coefficients drop? Maybe a few words of discussion could be added.

Minor comments:
- There is an issue with reference formatting, some appear as numbers, others as author, date.
- Why is monoicy called epigenetic sex determination?
- what species are monoicious on Figure 1? Please add color code in the legend.

Reviewer #2 (Remarks to the Author):

In this manuscript, the authors investigate the molecular basis and convergence of transitions from dioecy to monoecy by using RNA-seq and morphological data from four dioicous-monoicous species pairs. Although the evolution of separate sexes from hermaphroditic or monoecious ancestors has been extensively investigated, the opposite transition from separate sexes to co-sexuality has been comparatively understudied. The authors find that convergent changes in male-biased genes as a

result of selection and coding sequence evolution are the main drivers of transitions to monoecy. I found this manuscript and the findings presented here to be interesting and novel, and of high relevance to the growing understanding of the evolution of sex and asexuality. I have, however, a few comments and questions that I think should be addressed to improve the clarity of the manuscript.

General comments:

My main issue with the manuscript concerns the inferences drawn from the GO terms analysis. In my opinion, the evidence for convergence in sex-biased gene function is not very strong and the authors are overstating the results in Lines 165-171 and Lines 302-311. Biological processes related to 'microtubule', 'ion transport' and 'cilium' are not consistently enriched in male-biased genes across all dioecious species, as the authors state. At best, these biological processes are found among male-biased genes in two out of the four species, as seen in both Fig. 3C and Table S5. The same applies to the terms 'photosynthesis' and 'oxidation-reduction processes' that are enriched in female-biased genes of only one or two of the species. In addition, terms related to metabolism are found among both male-biased and female-biased genes of all species, and not just in female-biased genes as the authors state. There is also some inconsistency between results in Fig. 3C and Table S5. For example, in Fig. 3C male-biased genes of S. firma are enriched for 'cilium assembly', however this is missing from Table S5. The authors should revise the Results and Discussion sections, toning down statements such as "striking convergence in ... function" and "remarkable level of convergence in … function", and I would suggest perhaps even moving Fig. 3C to the supplementary material.

What do the authors think are the reasons for the large differences in the number of predicted genes and the number of expressed genes within some of the species pairs? I am referring in particular to differences in gene numbers between D. herbacea and D. dudresnayi and between S. firma and C. linearis (Table S1).

Related to my question above, the percentage of expressed genes is quite low for some of the species (Table S1). Could this be due to the stringent criteria for considering a gene as being expressed? How did the authors decide on the threshold of 5th percentile of TPM distribution, considering that other studies use a TPM > 2 threshold for calling a gene expressed? I wonder how much the choice of expression filtering influences downstream analyses such as the differential gene expression and the orthology analyses.

Fig 1E: I think with the exception of D. herbacea, the values on the x-axis are mixed up between the species. Mean cell length differences between the sexes should actually be ~1.01 for S. firma, ~4.65 for S. rigidula and ~12.59 for S. polyschides (based on the data in Table S3). The linear regression analysis should be redone with the correct values for each species.

Related to my point about the criteria for identifying expressed genes, S. rigidula has the lowest percentage of expressed genes and also the lowest number of sex-biased genes. I wonder how much this affects the correlation between phenotypic sexual dimorphism and the number of sex-biased genes. Does the correlation in Fig. 2E become significant if S. rigidula is removed from the linear regression analysis?

Specific comments:

In Lines 110-111 the authors say that genes were considered expressed if they had a TPM > 5th percentile counts across all genes in a minimum of 2 samples. However, in Lines 393-395 a minimum of 1 sample is mentioned. Which one is correct? If the former is true, does that mean that for the species with 2 replicate samples (S. polyschides and S. dermatodea), both samples needed to pass this threshold in order for a gene to be considered expressed?

Line 111-112: I do not think Fig. S2 should be referenced here as that figure relates to the GO enrichment analysis.

It would be useful to mention in the Fig. 1 legend which color represents species with dioicous systems and which represents those with a monoicous system.

Lines 122-123: The percentages of sex-biased genes for S. polyschides reported here differ slightly from those shown in Fig. 2A. For consistency, I suggest that the same method of calculating the percentage of male- and female-biased genes should be used throughout (either based on total number of expressed genes averaged across sexes, or based on total number of expressed genes within each sex individually).

Fig. 2B: Perhaps I am missing something, but I do not understand the purpose of highlighting in black the unbiased genes with a FC > 2. Those genes do not meet the sex-biased gene criteria and, if I am not mistaken, they are not really used in any of the analyses. I suggest representing these unbiased genes in gray points.

Table S3: For Gametophyte cell size, the ratio of female:male for S. firma should actually be 1.1 instead of 0.9, and for S. rigidula the ratio should be 1 instead of 0.

Lines 176-177: Maybe the authors meant to refer to Fig. 4A here instead of Fig. 2A?

Lines 374-376: Why were two different transcriptome assemblers used?

Lines 432-433: In these cases, which of the duplicated sequences was chosen and why?

Reviewer #3 (Remarks to the Author):

This study explores gene expression changes associated with the transition from unisexuality to co-sexuality in four phylogenetically distant and independent pairs of brown algae. In contrast to animal systems, the extent of sex-biased gene expression in brown algae was not correlated with phenotypic differences between female and male gametophytes. Sex-biased genes evolved fastest when male-biased and faster than genes with unbiased expression and their turnover rate was high, in notable contrast to their predicted functions. A key finding is that transitions to co-sexuality involved gene expression shifts mediated by natural selection. Together with the fact that genes with similar predicted functions were affected during the different transitions suggests that common drivers underlie the observed expression changes. Also, gene expression profiles of co-sexual species were found to be more similar to those of females than to males of related dioicous species, suggesting that they have arisen from ancestral females.

In general, I find this study very well presented and enjoyed reading it. My comments are hence minor and primarily aim to improve the accessibility of the topic.

In general, I hope that the difference between the terms ‘dioecy’ and ‘dioicy’ (e.g. line 87), as well as between ‘dioecious’ and ‘dioicous’ etc. can be explained early on and in a bit more detail. Else, readers may suspect typos or be confused. Also, while ‘dioecy’ is used correctly in the context of plants, I am confused why it is also used in context of brown algae on line 297.

In-text citations are given inconsistently, with some citations using numbers and others a more conventional format with author names and year of publication.

While do authors do a good job in explaining what they do and what they find (paragraph starting on line 87), the study is overall very descriptive. It would be interesting to learn early on what expectations exist and/or what major hypotheses can be tested with this exciting dataset.

The authors find no correlation between the level of sex-biased gene expression and the degree of phenotypic sexual dimorphism in brown algae. This led them to ask whether this is a consequence of “a lower degree of sexual selection in the brown algae”. What evidence is there in these organisms for sexual selection? This aspect is taken up a bit later in the discussion but without providing much detail. Here, I would hope for a more detailed discussion. Also, are there possible alternative drivers of sex-biased gene expression evolution in brown algae? Evidence for selection driving sex-biased gene expression evolution is strong – what are the underlying factors and drivers?

Along the same line I wonder whether levels of sex-biased gene expression are correlated with size of U and V chromosomes, specifically the sizes of (or numbers of genes in) the SDR?

A particularly interesting finding in my opinion is that male-biased genes are the main drivers of gene expression changes during the transition to monoicy in brown algae. Do I understand correctly that these genes are in many cases down-regulated in the monoicous relative?
In this context, I struggle with Fig. 4C. I do not fully understand the two panels labelled ‘Male’ and ‘Female’. Also, these results receive little attention in the results section, despite their prominence in the discussion.
Also, I fail to see the reminder of differences between male and female gamete size mentioned in the legend to Fig. 4C (“A reminder of differences between male and female gamete size within species pair is sketched below the x-axis.”).

While the study is focusing on gene expression changes associated with the transition from unisexuality to co-sexuality, one does not learn how such transitions occur – or at least, what the relevant genetic difference(s) between unisexual and co-sexual species is. This information may help readers following this study.

*******************END*******************

naturereasearch

Author Rebuttal to Initial comments

Reviewers' comments:

Reviewer #1 (Remarks to the Author):

Cossard et al provide a detailed analysis of the evolution of gene expression levels in eight species of brown algae sampling 4 independent transitions from separated sexes (dioicy, the ancestral state) to cosexuality. Their main result, I find, is that the evolution of cosexuality is accompanied by a convergence in gene expression among the four independently evolved cosexual species. Figure 4E remarkably illustrates this finding. Other noteworthy findings are that (i) the amount of sex-biased genes in dioicous species is not correlated to the level of sexual dimorphism, which confirms results obtained on the plant genus Leucadendron and add up to the potential distinction between animals and plants+algae, (ii) cosexuals species expression resembles female expression from dioicous species, suggesting that maybe cosexual species have evolved from females, (iii) sex-biased gene expression has a high turnover.
The manuscript is well written. I however have a few concerns about clarity (mainly regarding Figures) and one additional analysis that I would like to suggest.

Are monoicous species UU? If this is unknown the authors could at least discuss this question. This would give some more perspective to the expression resemblance between females and cosexuals.

Re: This is a very interesting comment. Monoicous species in these brown algae express sex during the haploid stage of the life cycle, and, strictly speaking, they do not have sex chromosomes (the same haploid genotype can produce male or female structures) and they have only one copy of the genome (haploid). A sentence in the introduction explains: "*Organisms with haploid sex determination may also produce male and female sexual structures in the same (co-sexual) individual (monoicy)* [29,30]". The reviewer is correct that if the monoicous species are derived from a U (female)- individual then they would be expected to have an 'ex-' U sex chromosome (and they presumably would have more resemblance with a female individual from the closest dioicous species). However, it would be unlikely that the monoicous gametophytes would be UU because monoicous species are also haploid. While we discuss the resemblance in terms of gene expression between dioicous and monoicous species in the current paper, the genomic analysis and the investigation on the fate of the U chromosome in a monoicous context is however not the focus of the present paper.

Throughout the manuscript, sex-biased expression is interpreted in terms of adaptation (for example Lines 241-243). However, Scharmann et al 2021 Biorxiv very convincingly show that sex-biased expression could be the result of random expression evolution under low selective pressures for non pleiotropic genes. I suggest the authors do a similar analysis to Scharmann et al 2021 Biorxiv and look at the rate of expression evolution of genes orthologous to sex biased genes in species where the genes are not sex biased.

Re: If we understand correctly, the reviewer would like us to test if the sex biased genes tend to evolve their expression levels faster than unbiased genes prior to the transition to monoicy (i. e, already in the ancestral dioicous context) as in Scharmann and colleagues. We thank the reviewer for the suggestion and agree this is an interesting analysis. We have therefore followed (Scharmann et al. 2021) and measured the rates of gene expression evolution using phylogenetic independent contrast method (PICs). Briefly, we performed the analysis as follows: we looked at the orthologs across the four dioicous species where one ortholog was sex-biased and the other three were unbiased. Then, we excluded the ortholog that was sex biased and measured the rate of expression evolution of the three-remaining unbiased orthologs. Finally, we compared the expression evolution between the three unbiased orthologs

versus orthologs that are unbiased across the four species. Our data set allowed us to explore PICs among 5914 orthologous genes across the four dioicous species, which had either no bias in any of the species (unbiased, 3845 orthologs) or sex-biased in a single species (2059 orthologs). Note that we could not compute PIC for orthologous genes that were sex-biased in more than one species as our phylogenetic tree only has four tips (four species).

We found that the mean difference of absolute standardized PIC between sex-biased genes (SBG) and unbiased genes (UnB) was 0.060 and that this difference was slightly significant (Mann-Whitney rank test P = 0.0495). It appears therefore that, similarly to Leucodendron and the European grayling (Scharmann et al. 2021; Papakostas et al. 2014) the faster gene expression evolution of SBG in brown algae may be due to lower level of functional constraints experienced by those genes prior to the evolution of sex-bias, possibly related to their lower level of pleiotropy. In the case of brown algae, the difference of expression evolutionary rates was relatively low in comparison to what has been found across Leucadendron genus, where the authors suggested that the emergence of sex-biased gene expression was facilitated for those genes experiencing lower constraints on their expression level. This means that in the case of brown algae, sexual selection may also play some role in the evolution of sex biased gene expression.

These new results therefore do not affect the overall conclusions of our paper, and actually enrich the manuscript. We however agree with the reviewer that we should nuance our claims concerning the role of sexual selection in the evolution of sex-bias among dioicous species because the PIC analysis shows that it is likely that these genes were already under lower constrain before evolving sex-biased expression. Indeed, both putative selective pressures as well as a lower degree of pleiotropy of sex-biased genes may bring about the observed patterns of sex-biased gene expression evolution. Accordingly, we added a paragraph about these new analyses in the revised version of the manuscript:

"We also asked whether sex-biased gene expression emerged in dioicous species as a result of random expression evolution under low selective pressure for non-pleiotropic genes[36] or rather as a consequence of sexual selection. To distinguish between these two possibilities, we computed phylogenetic independent contrasts (PICs) of sex-biased genes across species in which those genes are not sex-biased versus unbiased genes (Figure S2). We found that PICs differed slightly between unbiased genes and orthologs of sex-biased genes in species in which those genes are not sex-biased (Mann-Whitney Rank test P = 0.0495). This result indicates that genes that evolved sex-bias may have done so because they already experienced low constrains on their expression levels, possibly due to lower pleiotropic expression patterns [36,39] although we cannot exclude that sexual selection may be also involved in the emergence of sex biased gene expression in brown algae." (line 174-183)

We have also modified the text in the discussion, in the section 'Sex-biased genes have high turnover but exhibit functional convergence' with the addition at the end of the first paragraph of the following paragraph:

'Interestingly, our study suggests that sex-biased expression may have emerged on genes that were experiencing lower selective constraints on their expression level, possibly due to lower pleiotropy rather than due to the sole effect of sex-specific selection occurring after the evolution of separate sexes. A similar situation has been described recently in plants [26] and animals [32]." (line 325-328)

The reviewer may note that our study diverges markedly from that of Scharmann and colleagues, who sampled a wide range of dioecious plant species in order to investigate the origin of sex-biased gene expression. In our study, we are focusing rather on one specific evolutionary transition, namely the transition from dioicy to monoicy in brown algae. The calculation of Δx takes into account the variation of expression level across samples in the dioicous species, so that the over-representation of sex-biased genes among genes with $|\Delta x| > 1$ should rather reflect the effect of the relaxation of sexual selection

occurring in the dioicous species and directional selection acting on their expression level. In this case, the statement mentioned by the reviewer in lines 284-286 ("*Taken together, our observations indicate that shifts from dioicy to monoicy involved modifications to transcriptional patterns (expression divergence), mostly in male-biased genes, that were likely driven by selection but also coding sequence evolution.*") remains correct in our opinion.

Finally, we have updated the Methods section to include the description of this new analysis: *"We computed absolute standardized phylogenetic independent contrasts (PICs) among dioicous species, using the ape package in R. Mean PICs were compared, using Mann-Whitney Rank tests, between non-biased genes and sex-biased genes with their expression measured in species in which they were not sex-biased." (lines 477-479)*

Were Figure 2E correlations corrected for phylogeny using PGLS?

Re: We thank the reviewer for the comment. These correlations had not indeed been corrected for phylogeny on the figure presented in the original manuscript. As suggested by the reviewer, the new version of the manuscript presents now a fit of the linear regression using phylogenetic generalized least squares method (PGLS) as implemented in the package nlme in R (Pinheiro et al. 2013). This analysis did not change our initial results, the correlations were still not significant. We reported the slopes and linear regression using PGLS on on the new version of the figure. Moreover, we replaced multiple r-square values by adjusted r-square values out of simple linear regression on these data. We have added the details of this analysis in the method section as follows: " (…) *To investigate the relationship between phenotypic sexual dimorphism and extent of sex-biased expression, phenotypic dimorphism was regressed against the fraction of sex-biased genes in R, corrected for phylogeny using pylogenetic generalized least square method as implemented in the nlme R package [60]" (lines 439-442).*

The legend of Figure 2E has also been modified as follows: *"Linear regressions of the fraction of female- and male-biased genes (in red and blue, respectively) among the mean number expressed genes across both sexes, against the mean difference in cell length recorded between the sexes (in µm), in the four dioicous species investigated. Linear regression were fitted through phylogenetic generalized least square method implemented in the R package "nlme". We report values of adjusted r^2 calculated with ANOVA." (Lines 545-549)*

Lines 169-171: I don't find the result of convergence in sex-bias expression striking in dioicous species because for example photosynthesis and oxidation/reduction processes appear in only 2 of the 4 species in Figure 3C. I did not check all terms but maybe the authors could find a more convincing statistical way to say that the terms are non randomly found in common in the different species? What is the chance given the number of GO terms and genes that one term is found in common in two species?

Re: The terms related to photosynthetic processes (chlorophyl, photosynthesis) are enriched in female-biased genes in three out of four species, the terms related to oxidation(-reduction) and metabolism are enriched in female-biased genes for all four species, the terms related specifically to carbohydrate metabolism are enriched in female-biased genes of three species. Functions related to cilia, flagella, microtubule-related processes are enriched in male-biased genes for all four species. We believe that this result is important because there are very few (almost no) sex-biased genes in common among these four species. Therefore, it appears that the functions of these different sex-biased genes do converge to some extent (in the sense that these genes were unbiased in the ancestor of the four species). To increase the clarity and highlight the similar GO terms across the different species, we have now bolded the common terms in Table S5.

However, we take on the suggestion of the reviewer and we have down-stated the sentence describing this result and replaced it by the following:

"(...)*Conversely, the sets of female-biased genes of all or the majority of the studied species were enriched for GO terms related to photosynthesis/chlorophyl processes, metabolism and oxidation/reduction (Figure 3C, Table S5).Taken together, our results indicate that whilst the different species do not share the same sex-biased gene set, males and females across these brown algae display a considerable level of convergence in terms of sex-biased gene functions." (line 168-173).*

Concerning the statistical tests that the reviewer asks: As GO enrichment analysis is done on the GO graph / GO network for each category (e.g. molecular function, biological process, cell compartment), but independently for each species, we originally employed classic GO enrichment analysis (Fisher's exact tests) to test whether the set of genes selected in our sample is on average more abundant and "enriched" in a particular molecular function, biological process, cell compartment than would be expected by looking at all genes or a background set of genes., We discussed with biostatistician colleagues about possible ways to quantify the significance of the convergence in terms of GO terms across several species in order to address the comment of the reviewer about statistical tests. We have performed a Benjamini-Hochberg correction of individually retrieved *P*-values to account for multiple testing. This was done in R by applying the p.adjust() function on the vector of individual p-values. The corresponding corrected p-values account for false discovery rate, so that the enrichment of the same GO term (or 'keyword') across several species (if we assume that they share functional homology that can be confirmed by GO-term mappings through sequence identity) reflects a common enrichment in similar functions.

The result of this test (FDR correction using Benjamini-Hochberg multiple-testing correction) on the P-values retrieved for each species did not change the original results reported in the previous version of the manuscript. We have added the adjusted p-values to Table S5. In other words, we can say that when the same GO term-related function arises from independent significant Fischer exact tests across the studied species, we are confident that it reflects common terms that are enriched in all species. Note also that we have anyway toned down our sentences related to this matter (please see above) and we remain 'qualitative', in the sense we do not try to quantify the 'level of convergence', and we simply report that the same or similar GOterms arise in several of the studied species.

Same Line 303: I wouldn't speak about remarkable convergence because it seems to me that just a few sex-biased genes involved in a similar function are enough for the term to appear in multiple species. So this does not mean that all sex-biased genes are involved in convergent functions, but rather that at least a few of them are.

Re: We agree with the reviewer that not all sex biased genes among the four species are involved in convergent functions, but at least some of them are, and this result is coherent with the fact that we think that these species have mainly primary sexual dimorphism, related mainly to their primary functions of producing a male and a female gamete (in contrast to animals where many sex biased genes are involved in many and complex phenotypic traits). We also agree with the reviewer that this result is not that surprising because these functions are probably related to primary sexual dimorphism, so that males produce a lot of male gametes (with flagella) for example, and females grow bigger and invest in metabolic pathways. As explained above, we have down-toned the sentence in line 303 to take the reviewer comment in consideration and highlight the fact that a subset (and not all) of sex biased genes have similar predicted functions in the four species: '*Although the dioicous brown algal species studied here shared very few sex-biased genes, we found a considerable level of convergence in terms of sex-biased gene function, at least for a subset of sex biased genes.' (line 329-331).*

Finally, we also deleted the adjective 'remarkably' in the abstract related to the convergence of gene functions.

I don't understand FIgure 3A, what intersect is it representing?

Re: Figure 3A represent the visualization of the intersections between orthologous sets as a matrix. We used a R package (UpSetR) as an alternative way to visualize the intersect between six groups (Conway et al. 2017), which is clearer, in our opinion, than an over-complicated Venn diagram with 6 groups.

The rows represent the sets (PSO, for Pairwise Single-copy orthologs, DSO for dioicous Single-copy orthologs, and ASO for All single-copy orthologs) and the columns represent their intersections. The bar plot represent the number of single copy orthologs shared by different intersections. List of all possible overlaps between species is marked in the rows on the left. Connected dots under each bar indicate which of the species overlaps are affected.

If we consider for instance the line 'S.fir-C.lin', we can see that we find a total of 8257 single copy orthologs between Sphaeriotrichia and Chordaria, out of which a set of 4125 orthologs are found within PSOs, 3211 are also found in the DSO set, 899 are also members of both the DSO and ASO gene sets and, finally, 22 orthologs are present in the ASO (all-single copy orthologs) only.

We have added more information on the new Figure 3A x and y axis to improve clarity, and also added more information in the figure legend to improve clarity.

I would like to see Figure 4A for unbiased genes, just to check for any potential expression normalization issues

Re: The new version of the manuscript includes a new figure (Figure S2) with the plots corresponding to the unbiased genes, as requested.

In Figure 4A, FBG in S. rigidula don't look very female biased. Even the ns symbol says there is no difference between F and M expression for these genes. Same for MBG in these species. There seems to be an issue there. If these genes were identified as sex-biased with two-fold change why do they have ns expression difference on Figure 4A?

Re: We thank the reviewer for this comment. This is a misunderstanding caused by us not being clear enough in the original manuscript. We have verified all the data and analysis, and we confirm that the genes were indeed identified as sex-biased with a 2-fold change difference between sexes using the dioicous species dataset only. In Figure 4A, however, the dataset that is represented is composed solely of genes that are 1:1 single-copy ortholog in the corresponding monoicous species. In other words, the data that is plotted represents a subset of the sex-biased genes (SBG) out of all SBG inferred in dioicous species. While sex-bias is inferred for each gene separately, the statistics in Figure 4A compare the whole distribution of expression levels across all genes per bias, which may explain the non-significance between male and females for the gene set calculated in the Sphacelaria-Halopteris species pair.

Moreover, note that contrary to the 'within dioicous-species' analysis of sex-biased gene expression, the analysis comparing monoicous versus dioicous species is normalized across male, female and monoicous samples so that the expression patterns do not reflect the sex-bias inferred within dioicous species.

We have added a note in the legend of the figure to emphasize that the SBG represented in the figure are a subset (1:1 orthologs) of the SBG and to highlight that the comparison focus on monoicous vs dioicous species. ". *Note that only the sex-biased genes with a single-copy ortholog in the corresponding monoicous specie are displayed in the plots (in other words, the SBG represented in the plots are a subset of the SBG identified within each dioicous species).* " (line 561-564)

Are statistical tests paired in Figure 4A across genes? The statistical test should be paired because gene expression in females is dependent on gene expression in males.

Re: We are not sure to fully understand the comment of the reviewer concerning the statistical tests performed for Figure 4A. Expression levels are measured individually per sample, before being averaged per sex. As a consequence, the levels of gene expression in males and females are independently measured. We acknowledge that the categories (female-biased genes, male-biased genes, non-biased genes) are based on the within species analysis that compares male to female expression. However, the level of expression of a given gene across one sex does not depend on the expression level in the opposite sex. Finally, by calculating the levels of expression as transcripts per millions (TPMs), we are able to compare the male, female and monoicous samples directly. Note that similar analysis has been performed in (Parker et al. 2019; Hollis et al. 2014), and these studies do not use paired-test across species to compare MBG vs FBG vs unbiased genes.

Lines 181-192 When looking at significance levels in Figure 4A the conclusion is that monoicous expression is often significantly DIFFERENT from female expression. Looking at bars it is true that monoicous expression seems closer to female expression than to male expression but this is very descriptive rather than quantitative. I think in this paragraph the authors should not conclude that monoicous expression is closer to female, this conclusion rather comes from Figure 4B.

Re: Indeed, the reviewer is correct, and this is a conclusion that we do not reach in this paragraph but rather in the following one, which actually describes the results of the Pearson coefficients. In the paragraph the referee is mentioning we simply describe, as the referee correctly points out, the difference in the distribution of gene expression levels between males, females and monoicous individuals for sex-biased genes separately. We see that, overall, the level of expression of male-biased genes is downregulated in the monoicous species and their level of expression is more similar to that of females of the corresponding dioicous species.

Line 187 the expression levels are significantly different between herbacea females and the corresponding monoicous species, please rephrase.

Re: We thank the reviewer for noticing this; it is indeed a mistake in the name of the species. This has been corrected.

Figure 4B is difficult to interpret. I would favor a less condensed representation because I don't understand where is the pearson coefficient between dioicous females and monoicous and where is the pearson coefficient between dioicous male and monoicous. All pearson coefficients should be shown separately for males and females and also for different gene sets (MBG, FBG and unbiased genes) The Figure only shows sex-biased genes in females and males but these gene sets have different male and female expression. I might have misunderstood completely Figure 4B, in which case a clarification would be welcome.

Re: The figure represents male versus female similarity indexes in relation to the monoicous expression profiles. The point of separating gene sets between sex-biased genes (SBG) and unbiased genes (in black) is to highlight the fact that SBG have more similar expression profiles between dioicous and monoicous species than unbiased genes. We propose to add two supplemental figures that show the data in a less condensed fashion as the reviewer suggests. We therefore provide as new Figure S4 the Pearson coefficients separately for males and females and as new Figure S5 the Pearson coefficients separately for the different gene sets (MBG, FBG and unbiased genes). We would prefer to keep these figures as supplement, and increase clarity of the main figure by adding more information in the legend the figure, but if the reviewer and editor prefer, we can of course completely replace the main figure by

these less condensed supplemental figures. We have also modified the legend to increase clarity and refer the reader to the two supplemental figures.

Figure 4C has two panels, one for male and one for female. Was delta x computed separately for dioicous male and female expression? This was very unclear from the methods and main text and should be clarified.

Re: Δx was calculated separately by sex, averaged separately across males, females and monoicous individuals, based on log2(TPM+1). The graph shows the proportion of orthologs with an average $|\Delta x| > 1$. This point was indeed unclear in the original materials and methods. We have modified the methods in the new version of the manuscript to includes the following: "*ΔX was computed separately for females and males of the dioicous species, and for male-biased genes, female-biased genes and unbiased genes also separately. Orthologs with $|\Delta x| > 1$ and a minimum expression fold-change between sister species of 1.5 were considered to have had a significant evolutionary expression shift. Fisher exact tests were computed to detect whether female-biased genes (FBG) and male-biased genes (MBG) were more likely to show an absolute value of $\Delta X > 1$ compared to unbiased genes." (line 451-456)*

Finally, we also modified Figure 4C so that: i) it also shows the values for unbiased genes and (ii) it represents the % of orthologs within each gene set and not over the total number of orthologs as it was in the first version of the manuscript. We have detailed this information in the legend accordingly.

Generally speaking, the Figures would benefit from additional color legends directly on the graphs and more detailed axis names because many graphs cannot be understood without reading the legend.

Re: The new version of the manuscript has taken in consideration this comment, and we have added more information in the legends and in the figures. We have also added supplementary figures to help increase clarity of the results description. We hope the reviewer is satisfied with our effort to increase clarity of the figures.

Line 209-211: Could this be due to a normalization issue? When many genes have sex-biased expression they affect other genes expression because they take up a high proportion of transcripts per million, making pearson coefficients drop? Maybe a few words of discussion could be added.

Re: We believe the reviewer refers to the sentence "*We also noted that the highest similarity indexes for within species pairs were found for the species with the lowest level of sex-biased gene expression (S. rigidula), and the lowest similarity was observed for S. firma, the species with the highest level of sex-biased gene expression*". Indeed, we do not have currently an explanation for this observation, but it is unlikely that this is due to normalization issues, as we have performed normalization of all data before differential expression analysis, as well as before performing comparison within species pairs (i.e., on PSO). In an attempt to further explore this result, we checked if this could be due to the fact that *S. firma* has a high proportion of sex specific genes, visible in Figure 2A – however this is unlikely to be the explanation because very few sex-specific genes are represented in the PSO set. We have added a sentence on the results section that mentions this additional test "*The relatively high proportion of sex-specific genes present in S. firma (Figure 2A) is unlikely to be the cause of the observed low similarity, because only 29 (0.49%) of the sex-specific genes are among the PSOs used for the Pearson similarity analysis.*"(line 226-228). Unfortunately, we cannot currently offer an interpretation of this result without being utterly speculative, so we would prefer not to add any discussion on this matter, and simply report the result.

Minor comments:
- There is an issue with reference formatting, some appear as numbers, others as author, date.

Re: The format of the references has now been checked and updated.

- Why is monoicy called epigenetic sex determination?

Re: The term 'epigenetic' was originally used because in monoicous (and monoecious) species sexes are not determined genetically but 'epigenetically' or 'developmentally' in the sense that there are no genomic differences between males and females (Pannell 2017; Beukeboom and Perrin 2015). Monoicy is the sexual system in which both sexual functions are expressed within the same individual, so that sex-determination leading to the production of male and female sexual structures occurs within the same individual. To avoid misunderstandings, we have however removed the term 'epigenetic' and we have also added more details on the definition of the terms monoicy, monoecy, dioicy and dioecy.

"The terms 'dioicy' (i.e., separate sexes during the haploid phase of the life cycle, as opposed to 'dioecy' where separate sexes occur in the diploid phase) and monoicy (i.e, co-sexuality during the haploid phase of the life cycle, as opposed to 'monoecy' where co-sexuality occurs in the diploid phase) are used to describe the sexual systems of these organisms [26]. " (lines 59-62).

- what species are monoicious on Figure 1? Please add color code in the legend.

Re: The legend was modified as following "Dioicous species (D) are marked in brown and monoicous species (M) in black.

Reviewer #2 (Remarks to the Author):

In this manuscript, the authors investigate the molecular basis and convergence of transitions from dioecy to monoecy by using RNA-seq and morphological data from four dioicous-monoicous species pairs. Although the evolution of separate sexes from hermaphroditic or monoecious ancestors has been extensively investigated, the opposite transition from separate sexes to co-sexuality has been comparatively understudied. The authors find that convergent changes in male-biased genes as a result of selection and coding sequence evolution are the main drivers of transitions to monoecy. I found this manuscript and the findings presented here to be interesting and novel, and of high relevance to the growing understanding of the evolution of sex and asexuality. I have, however, a few comments and questions that I think should be addressed to improve the clarity of the manuscript.

General comments:

My main issue with the manuscript concerns the inferences drawn from the GO terms analysis. In my opinion, the evidence for convergence in sex-biased gene function is not very strong and the authors are overstating the results in Lines 165-171 and Lines 302-311. Biological processes related to 'microtubule', 'ion transport' and 'cilium' are not consistently enriched in male-biased genes across all dioecious species, as the authors state. At best, these biological processes are found among male-biased genes in two out of the four species, as seen in both Fig. 3C and Table S5. The same applies to the terms 'photosynthesis' and 'oxidation-reduction processes' that are enriched in female-biased genes of only one or two of the species. In addition, terms related to metabolism are found among both male-biased and female-biased genes of all species, and not just in female-biased genes as the authors state.

Re: We thanks the reviewer for his positive view of our work. The new version of the manuscript has down-toned the statements related to convergence of sex-biased function as requested (see also response to Reviewer 1 comments). However, we would still argue that GO terms related to photosynthetic processes (chlorophyl, photosynthesis) are enriched in female-biased genes (FBG) in 3 out of 4 species, the terms related to oxidation(-reduction) and metabolism are enriched in FBG for all four species, the terms related specifically to carbohydrate metabolism are enriched in FBG for 3 out of 4

species. Functions related to cilia, flagella, microtubule-related processes are enriched in male-biased genes (MBG) for all four species. Overall, therefore, we believe there is a considerable amount of similar terms across species that are highlighted by the statistical analysis of TopGO. We believe that this result is important, especially in association to the fact that there are very few (almost no) sex biased genes in common among these 4 species. So, it appears that the functions of these different SBG converge to a certain degree (in the sense that these genes were unbiased in the ancestor of the 4 species). We hope the reviewer agrees with our interpretation.

We have nevertheless considered the suggestions of the reviewer, and we have toned down the sentences across the manuscript, as suggested, and removed strong adjectives when mentioning this result ("striking", "remarkable"). For example:

"*We detected significant enrichment of GO terms for biological processes related to 'microtubule', 'ion transport' and 'cilium' consistently for male-biased genes across dioicous species. Conversely, the sets of female-biased genes of all or the majority of the studied species were enriched for GO terms related to photosynthetic/chlorophyl process, metabolism and oxidation/reduction (Figure 3C, Table S5). Taken together, our results indicate that whilst the different species do not share the same sex-biased gene set, males and females across these brown algae display a considerable level of convergence in terms of sex-biased gene functions.*"(line 166-173).

To more easily visualize the similarity across species in terms of GOterms we have highlighted in bold the terms that are common among species in Table S5.

There is also some inconsistency between results in Fig. 3C and Table S5. For example, in Fig. 3C male-biased genes of S. firma are enriched for 'cilium assembly', however this is missing from Table S5.

Re: This has been corrected, and we thank the reviewer for spotting this. Table S5 was actually presenting the results of the GO terms for 'Molecular Functon' (and not 'Biological process' as in the Figure). We have replaced in Table S5 the correct GO terms matching the Figure 3C (now Figure S1).

The authors should revise the Results and Discussion sections, toning down statements such as "striking convergence in ... function" and "remarkable level of convergence in … function", and I would suggest perhaps even moving Fig. 3C to the supplementary material.
Re: This has been done. We have toned down the sentences as suggested by the reviewer, by removing words as 'remarkable' and 'striking' in respect to the convergence of gene function in sex-biased genes. AAs suggested, we have transferred Figure 3C to the supplemental material, it is now Figure S1.

What do the authors think are the reasons for the large differences in the number of predicted genes and the number of expressed genes within some of the species pairs? I am referring in particular to differences in gene numbers between D. herbacea and D. dudresnayi and between S. firma and C. linearis (Table S1).

Re: Concerning the differences between number of predicted genes, we believe these differences stem mainly from the various qualities of genomes used for gene prediction. The high number of predicted genes in *D. dudresnayi* may be due to duplicated sequences, pseudogenes or wrongly called genes by the predictor. Note, however, that when we look at the number of expressed genes, the discrepencies between species within pairs is strongly reduced, suggesting that superfluous genes are mainly mistakes from the prediction software. For instance in *D. dudresnayi*, out of 49820 predicted genes, we detect the expression of 27391 genes, which is a value more comparable to the ~14000 genes predicted for D. *herbacea*. In *Sphaerotrichia firma*, between 20207 and 24826 genes are expressed in females and males, respectively, while the predicted gene set is 30008 genes (Table S1).

natureresearch

Related to my question above, the percentage of expressed genes is quite low for some of the species (Table S1). Could this be due to the stringent criteria for considering a gene as being expressed? How did the authors decide on the threshold of 5th percentile of TPM distribution, considering that other studies use a TPM > 2 threshold for calling a gene expressed? I wonder how much the choice of expression filtering influences downstream analyses such as the differential gene expression and the orthology analyses.

Regarding the comment on criteria for threshold choice: we applied the threshold of the 5th precentile as a common practice in previously published RNAseq work (Lipinska et al. 2019). To the best of our knowledge there is not a standard rule for choosing thresholds, with different labs/different experiments using different approaches for calling a gene as 'expressed'. We and others believe a reasonable approach is to look at the distribution of the expression values and remove the lowly expressed (likely noise) genes (hence the 5th percentile). Usually there is variance in the distribution of TPMs among the species so the 5th percentile approach allows us to treat more uniformelly all the samples that are being compared, in contrast to setting an arbitrary threshold value. None of the threshold set this way exceeded TPM=2 so that we are confident that we apply a comparable criteria across all samples. Importantly, note that the DE analysis is not affected by this threshold because DEseq2 uses the raw read counts and not the TPMs. Non-expressed genes (TPM=0) across replicates in a given species are also removed. The threshold of TPM>5th percentile is only applied when we do the comparisons between orthologous gene sets.

We have nevertheless tested in a subset of data if the choice of a threshold of TPM>2 instead of TPM>5th percentile would fundamentally change our results. As shown in the figure below, this is not the case, so we are confident that our choice of threshold method is valid.

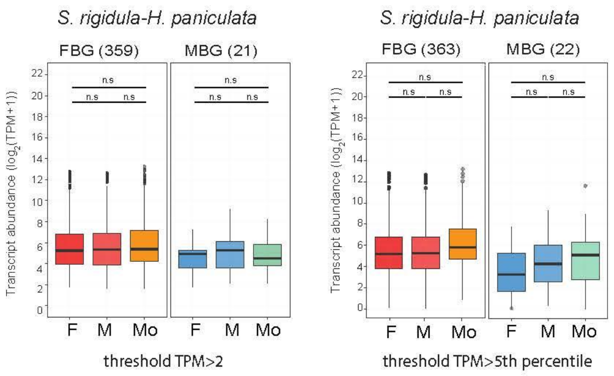

Fig 1E: I think with the exception of D. herbacea, the values on the x-axis are mixed up between the species. Mean cell length differences between the sexes should actually be ~1.01 for S. firma, ~4.65 for S. rigidula and ~12.59 for S. polyschides (based on the data in Table S3). The linear regression analysis should be redone with the correct values for each species.

Re: The reviewer is correct, labels of the right-hand legend in the figure were mixed up. This has been corrected. We also modified the figure following remarks of Reviewer #1, showing the linear regression fitted by PGLS. There was no change in the conclusions.

Related to my point about the criteria for identifying expressed genes, S. rigidula has the lowest percentage of expressed genes and also the lowest number of sex-biased genes. I wonder how much this affects the correlation between phenotypic sexual dimorphism and the number of sex-biased genes. Does the correlation in Fig. 2E become significant if S. rigidula is removed from the linear regression analysis?

Re: We thank the reviewer for this comment. We performed, as suggested, the linear regression removing the Sphacelaria species pair form the analysis. This did not lead to a significant correlation neither for the male-biased genes ($P = 0.431$ for a coeficient of -0.3578) or for the female-biased genes ($P=0.954$ for a coeficient of -0.01086). Therefore, there is no change in the results and conclusions, and we have retained this species in the analysis.

Specific comments:
In Lines 110-111 the authors say that genes were considered expressed if they had a TPM > 5th percentile counts across all genes in a minimum of 2 samples. However, in Lines 393-395 a minimum of 1 sample is mentioned. Which one is correct? If the former is true, does that mean that for the species with 2 replicate samples (S. polyschides and S. dermatodea), both samples needed to pass this threshold in order for a gene to be considered expressed?

Re: We thank the reviewer for detecting this typo. The description of the approach in the Material and Methods section is the correct one. We modified the text from ' in a minimum of 2 samples' to 'in at least one sample' (line 123).

Line 111-112: I do not think Fig. S2 should be referenced here as that figure relates to the GO enrichment analysis.

Re: The reviewer is correct; we have removed this reference to Figure S2 from the main text.

It would be useful to mention in the Fig. 1 legend which color represents species with dioicous systems and which represents those with a monoicous system.

Re: We added in the legend the color code for dioicous (D) and monoicous (M) species.

Lines 122-123: The percentages of sex-biased genes for S. polyschides reported here differ slightly from those shown in Fig. 2A. For consistency, I suggest that the same method of calculating the percentage of male- and female-biased genes should be used throughout (either based on total number of expressed genes averaged across sexes, or based on total number of expressed genes within each sex individually).

Re: We agree that a single measure of percentages of sex-biased genes should be used throughout the manuscript. We have therefore replaced values throughout the main text by percentages calculated over the mean number of expressed genes between males and females, as represented on Figure 2A.

Fig. 2B: Perhaps I am missing something, but I do not understand the purpose of highlighting in black the unbiased genes with a FC > 2. Those genes do not meet the sex-biased gene criteria and, if I am not mistaken, they are not really used in any of the analyses. I suggest representing these unbiased genes in gray points.

Re: If we understand correctly, the reviewer refers to the black points in Figure 2B. We included the points in black because we felt that this information could be useful to the reader. However the reviewer is correct to point that these genes, as they do not meet the critera for being considered sex-biased by DESEQ2, are not used in downstream analyses. We have therefore updated the figures in the new version of the manuscript and, following the suggestion of the reviewer, we represent now those genes as grey points.

Table S3: For Gametophyte cell size, the ratio of female:male for S. firma should actually be 1.1 instead of 0.9, and for S. rigidula the ratio should be 1 instead of 0.

Re: This is an unfortunate error, we thank the reviewer for pointing this out. The values have been corrected and there is no effect in the results.

Lines 176-177: Maybe the authors meant to refer to Fig. 4A here instead of Fig. 2A?

Re: This has been corrected.

Lines 374-376: Why were two different transcriptome assemblers used?

Re: Two of the assemblies used in the present study (*Chordaria linearis*, *Sphaerotrichia firma*) were performed by colleagues from our group in the framework of another project. Since these assemblies were of high quality and we wanted to remain coherent with the gene names and structure across the different projects, we used the already available datasets.

Lines 432-433: In these cases, which of the duplicated sequences was chosen and why?

Re: In the cases where a duplicated gene was present in one genome, the longest gene of the two was retained in the dataset of orthologs for downstream analysis. To clarify this point we have added the following sentence on the methods in the new version of the manuscript: '*In the later case, the longest duplicated sequence was retained for further analysis' (line 468-469).* A total number of nine orthogroups passed this criteria of alignment, all due to apparent duplication in the gene set of *Sphacealaria rigidula*.

Reviewer #3 (Remarks to the Author):

This study explores gene expression changes associated with the transition from unisexuality to co-sexuality in four phylogenetically distant and independent pairs of brown algae. In contrast to animal systems, the extent of sex-biased gene expression in brown algae was not correlated with phenotypic differences between female and male gametophytes. Sex-biased genes evolved fastest when male-biased and faster than genes with unbiased expression and their turnover rate was high, in notable contrast to their predicted functions. A key finding is that transitions to co-sexuality involved gene expression shifts mediated by natural selection. Together with the fact that genes with similar predicted functions were affected during the different transitions suggests that common drivers underlie the observed expression changes. Also, gene expression profiles of co-sexual species were found to be more similar to those of females than to males of related dioicous species, suggesting that they have arisen from ancestral females.

In general, I find this study very well presented and enjoyed reading it. My comments are hence minor and primarily aim to improve the accessibility of the topic.

In general, I hope that the difference between the terms 'dioecy' and 'dioicy' (e.g. line 87), as well as between 'dioecious' and 'dioicous' etc. can be explained early on and in a bit more detail. Else, readers may suspect typos or be confused. Also, while 'dioecy' is used correctly in the context of plants, I am confused why it is also used in context of brown algae on line 297.

Re: We thank the reviewer for the positive comments. The term dioecy is explained in the introduction, but we agree perhaps not with enough details. We have therefore rephrased the following sentences to more thoroughly explain the term: "*The terms 'dioicy' (i.e., separate sexes during the haploid phase of the life cycle, as opposed to 'dioecy' where separate sexes occur in the diploid phase) and monoicy (i.e, co-sexuality during the haploid phase of the life cycle, as opposed to 'monoecy' where co-sexuality occurs in*

the diploid phase) are used to describe the sexual systems of these organisms [26]. Genetic sex determination in dioicous organisms occurs during meiosis (and not at fertilisation as in XY and ZW systems), depending on whether spores inherit a U or V chromosome.(…)" (lines 59-64)

The mention of 'dioecy' on line 297 was a typo. We modified it to 'dioicy' in the main text.

In-text citations are given inconsistently, with some citations using numbers and others a more conventional format with author names and year of publication.

Re: This has been corrected.

While do authors do a good job in explaining what they do and what they find (paragraph starting on line 87), the study is overall very descriptive. It would be interesting to learn early on what expectations exist and/or what major hypotheses can be tested with this exciting dataset.

Re: We have followed the reviewer's advice and tried to clarify and highlight the hypothesis underlying the analysis in the new version of the manuscript "*Here, we explore multiple, repeated events of loss of dioicy (Figure 1) to investigate the molecular basis and level of convergence of the shifts to co-sexuality. We test the hypothesis that sexually dimorphic algae might be expected to have more sex-biased genes and, because dioicy is ancestral, we predicted that similar gene sets would be sex-biased across all the dioicous species. Contrary to our prediction, we demonstrate a lack of correlation between phenotypic sexual dimorphism and gene expression levels among dioicous brown algae. Ancestral state reconstruction indicated high turnover rates of sex-biased genes, yet independently recruited sex-biased genes shared similar functions across the species. To characterise the molecular changes associated with the evolution of monoicy, we then focused on modifications in gene expression patterns of orthologous genes that are specifically or preferentially expressed in haploid males and females of a dioicous species, when they function in a monoicous context. Male-biased genes were particularly concerned by both adaptive expression shifts and faster evolutionary rates associated with the transition to monoicy. Monoicous species displayed expression profiles that were more similar to those of the female of the closely related dioicous species than to the male. Finally, we identified an important amount of convergent gene expression changes associated with the emergence of co-sexuality, which were likely driven by selection."* (line 86-99).

The authors find no correlation between the level of sex-biased gene expression and the degree of phenotypic sexual dimorphism in brown algae. This led them to ask whether this is a consequence of "a lower degree of sexual selection in the brown algae". What evidence is there in these organisms for sexual selection? This aspect is taken up a bit later in the discussion but without providing much detail. Here, I would hope for a more detailed discussion. Also, are there possible alternative drivers of sex-biased gene expression evolution in brown algae? Evidence for selection driving sex-biased gene expression evolution is strong – what are the underlying factors and drivers?

Re: Because the aim of the study was not to investigate the evolution of sex biased gene expression but to focus on the transitions from separate sexes to hermaphroditism, we had originally chosen not to write a too extensive discussion about sexual selection and sex biased genes themselves, which we worried would divert the reader. We however agree that this aspect deserves more attention and we thank the reviewer for the suggestion. Also in response to Reviewer 1, the new version of the manuscript includes a section on the phylogenetic independent contrasts analysis, which offers an insight into the potential causes of sex biased gene evolution in the studied brown algal species. The analysis shows that sex-biased genes present higher variance in expression than unbiased genes revealing lower level of constrains that may have allowed the evolution of sex-bias expression. However, the difference was low and marginally significant, suggesting that sexual selection may also have driven the evolution of sex-

bias. These new results are now described in lines 187-196 and included in the discussion section (line 349-352).

In addition, we have, as suggested by the reviewer, elaborated in the discussion section about the main drivers of sex biased gene expression in brown algae. Indeed, evolutionary analysis performed previously on sex-biased genes in the model brown alga *Ectocarpus* shows they are evolving faster than unbiased genes and positive selection plays a role in this pattern, at least to a certain degree (Lipinska et al. 2015) We have therefore added the following sentence in the discussion. "(…) *Noteworthy, sex-biased genes found in male and female gametophytes of Ectocarpus showed more rapid rates of divergence across species (measured as dN/dS) compared to unbiased genes, and their accelerated evolution was at least partly attributed to positive selection 15. These observations suggest that sexual selection play a role in the evolution of sex-biased genes in brown algae, but may not be the only driver of sex-biased gene expression in this group of organisms." Line 313-318.*

Along the same line I wonder whether levels of sex-biased gene expression are correlated with size of U and V chromosomes, specifically the sizes of (or numbers of genes in) the SDR?

Re: This is an excellent question, and indeed this is something we are currently investigating. We have previously published data for the size of the sex determining regions (SDRs) within the U and V sex chromosomes for *Ectocarpus* and we are currently analysing datasets from genomes of several other brown algae to investigate the size of the sex locus and relate it to the level of sexual dimorphism. However, this is a long-term project requiring the generation of high-quality genome assemblies for multiple species and the data necessary for the analysis are not yet fully available. We therefore feel that describing the sex chromosomes across these brown algae falls out of the subject for this manuscript and we would prefer to restrain from discussing this here.

A particularly interesting finding in my opinion is that male-biased genes are the main drivers of gene expression changes during the transition to monoicy in brown algae. Do I understand correctly that these genes are in many cases down-regulated in the monoicous relative?

Re: We agree with the reviewer on this point. Figure 4C indicates that male-biased genes are down regulated in the monoicous species in comparison to the male ancestor. These genes are also more likely to be concerned by directional selection as the driver of these expression changes. Note however that when compared to their level of expression in females, the male-biased genes are still up-regulated in the monoicous compared to ancestral females. Our data suggests that the ancestor was a female that acquired male function genes, so this would explain why male-biased genes are more changing than female biased genes. Note that we cannot completely rule out that this effect is due to the fact that female-biased genes have more pleiotropic functions, in this case the changes would be less conspicuous in female-biased genes during the transition because these genes would have more broad functions.

In this context, I struggle with Fig. 4C. I do not fully understand the two panels labelled 'Male' and 'Female'. Also, these results receive little attention in the results section, despite their prominence in the discussion.

Re: The manuscript now includes extensive information in the results section about this figure: "*We computed Δx of the PSO sets, separately for each pair of species and reported the proportions of orthologs with an absolute $\Delta x > 1$, i.e., orthologs whose expression shift is attributable to directional selection (Table S11, Figure 4C). Depending on the species pair, between 10.8% and 40.1% of unbiased genes exhibited expression shifts attributable to selection ($|\Delta x|>1$) (Table S11). We then asked whether male- and female-biased genes were preferentially concerned by adaptive expression shifts during transitions to monoicy compared with unbiased genes. Figure 4C illustrates the proportion of orthologs*

with sex-bias displaying |Δx| > 1 (in other words, under putative directional selection) and how sex-biased genes are more likely to display |Δx| > 1 in comparison to the unbiased orthologs. Fisher's exact tests (asterisks in Figure 4C) showed that for three out of four species pairs, male-biased genes were indeed more likely to display |Δx|>1 than unbiased genes (Figure 4C). This was also the case for female-biased genes in S. polyschides-S. dermatodea pair of species (Fisher exact tests, P<2.2 x10-16 in both sexes) and D. herbacea-D. dudresnayi pair (Fisher exact tests, P = 3.9 x10-3 and P = 1.8 x10-5 in females and males, respectively). In S. polyschides-S. dermatodea and S. rigidula-H. paniculata, female-biased genes showed lower levels of adaptive evolution of expression compared with unbiased genes (Table S11, Figure 4C). Taken together, our observations indicate that male-biased genes preferentially exhibit a shift in expression during the transition to monoicy that may be explained by directional selection. (line 251-264):

We have also added the unbiased gene information within the figure, which hopefully makes it easier to interpret. We hope that the additional explanations and changes make the figure clearer. Also, note that the legend of the Figure presents an exhaustive description that complements the result section.

Also, I fail to see the reminder of differences between male and female gamete size mentioned in the legend to Fig. 4C ("A reminder of differences between male and female gamete size within species pair is sketched below the x-axis.").

Re: After discussion among authors, we decided not to include a reminder of sexual dimorphism of gamete size on this figure in the new version of the manuscript, as it is not particularly relevant here. We have removed the superfluous sentence from the legend of Figure 4C.

While the study is focusing on gene expression changes associated with the transition from unisexuality to co-sexuality, one does not learn how such transitions occur – or at least, what the relevant genetic difference(s) between unisexual and co-sexual species is. This information may help readers following this study.

Re: The aim of this study was to understand the changes at the level of gene expression that follow several independent transitions to monoicy, in particular in what concerns genes that are specifically expressed in male and female and how their patterns of expression are modified in a monoicous context. The genetic basis of the transitions (i.e., the differences between dioicous and monoicous species at the level of their genomes, or the genes that may be involved in the transitions) is a very interesting topic in itself as a stand-alone manuscript, but we believe it is out of the scope of the present manuscript. As mentioned above, we are currently analyzing datasets from genomes of several brown algae to investigate specifically the sex chromosomes and also to study the genetic/genomic consequences of transitions to co-sexuality across several transitions from dioicy towards monoicy. However, this is a long-term project requiring the generation of high-quality genome assemblies for multiple species and the data necessary for the analysis are not yet fully available.

References

Beukeboom L, Perrin N. 2015. *The Evolution of Sex Determination*. Oxford University Press.

Conway JR, Lex A, Gehlenborg N. 2017. UpSetR: an R package for the visualization of intersecting sets and their properties. *Bioinforma Oxf Engl* **33**: 2938–2940.

Hollis B, Houle D, Yan Z, Kawecki TJ, Keller L. 2014. Evolution under monogamy feminizes gene expression in Drosophila melanogaster. *Nat Commun* **5**: 3482.

Lipinska AP, Serrano-Serrano ML, Cormier A, Peters AF, Kogame K, Cock JM, Coelho SM. 2019. Rapid turnover of life-cycle-related genes in the brown algae. *Genome Biol* **20**: 35.

Lipinska, Cormier A, Luthringer R, Peters AF, Corre E, Gachon CMM, Cock JM, Coelho SM. 2015. Sexual dimorphism and the evolution of sex-biased gene expression in the brown alga *Ectocarpus*. *Mol Biol Evol* **32**: 1581–1597.

Pannell JR. 2017. Plant Sex Determination. *Curr Biol CB* **27**: R191–R197.

Papakostas S, Vøllestad LA, Bruneaux M, Aykanat T, Vanoverbeke J, Ning M, Primmer CR, Leder EH. 2014. Gene pleiotropy constrains gene expression changes in fish adapted to different thermal conditions. *Nat Commun* **5**: 4071.

Parker DJ, Bast J, Jalvingh K, Dumas Z, Robinson-Rechavi M, Schwander T. 2019. Sex-biased gene expression is repeatedly masculinized in asexual females. *Nat Commun* **10**: 4638.

Pinheiro J, Bates D, DebRoy SS, Sarkar D. 2013. Nlme: linear and nonlinear mixed effects models. *R Package Version 31-110* **3**: 1–113.

Scharmann M, Rebelo AG, Pannell JR. 2021. High rates of evolution preceded shifts to sex-biased gene expression in *Leucadendron*, the most sexually dimorphic angiosperms. *bioRxiv* 2021.01.12.426328.

Yamamoto K, Hamaji T, Kawai-Toyooka H, Matsuzaki R, Takahashi F, Nishimura Y, Kawachi M, Noguchi H, Minakuchi Y, Umen JG, et al. 2021. Three genomes in the algal genus Volvox reveal the fate of a haploid sex-determining region after a transition to homothallism. *Proc Natl Acad Sci U S A* **118**.

Decision Letter, first revision:

6th October 2021

*Please ensure you delete the link to your author homepage in this e-mail if you wish to forward it to your co-authors.

Dear Dr Coelho,

Your manuscript entitled "SELECTION DRIVES THE EVOLUTION OF CONVERGENT GENE EXPRESSION CHANGES DURING TRANSITIONS TO CO-SEXUALITY IN HAPLOID SEXUAL SYSTEMS" has now been seen by two of the original reviewers, whose comments are attached. The reviewers continue to have a number of serious concerns which will need to be addressed before we can offer publication in Nature Ecology & Evolution. We will therefore need to see your responses to the criticisms raised, along with a revised manuscript, before we can reach a final decision regarding publication. Please keep in mind that, especially as the reviewers were not convinced that some of their concerns from the original round of review have been resolved, we will be reluctant to send the manuscript back to review unless we feel that the next set of revisions are more likely to resolve all their concerns.

While we understand that you previously disagreed with Reviewer #1's request for paired statistical tests, this recommendation does seem reasonable to us. We do not believe that Reviewer #1 meant to always pair male and female expression, but rather that in each comparison, the tests should be based on differences in expression of specific genes, rather than differences in the full distribution of gene expression across genes (i.e. the genes are paired in the analysis). My apologies if we have misunderstood your explanation and please let me know if it would be helpful to discuss further before you submit your revised manuscript.

We therefore invite you to revise your manuscript taking into account all reviewer and editor comments. Please highlight all changes in the manuscript text file.

We are committed to providing a fair and constructive peer-review process. Do not hesitate to contact us if there are specific requests from the reviewers that you believe are technically impossible or unlikely to yield a meaningful outcome.

When revising your manuscript:

* Include a “Response to reviewers” document detailing, point-by-point, how you addressed each reviewer comment. If no action was taken to address a point, you must provide a compelling argument. This response will be sent back to the reviewers along with the revised manuscript.

* If you have not done so already please begin to revise your manuscript so that it conforms to our Article format instructions at http://www.nature.com/natecolevol/info/final-submission. Refer also to any guidelines provided in this letter.

* Include a revised version of any required reporting checklist. It will be available to referees (and, potentially, statisticians) to aid in their evaluation if the manuscript goes back for peer review. A

revised checklist is essential for re-review of the paper.

Please use the link below to submit your revised manuscript and related files:

[REDACTED]

Note: This URL links to your confidential home page and associated information about manuscripts you may have submitted, or that you are reviewing for us. If you wish to forward this email to co-authors, please delete the link to your homepage.

We hope to receive your revised manuscript within four to eight weeks. If you cannot send it within this time, please let us know. We will be happy to consider your revision so long as nothing similar has been accepted for publication at Nature Ecology & Evolution or published elsewhere.

Nature Ecology & Evolution is committed to improving transparency in authorship. As part of our efforts in this direction, we are now requesting that all authors identified as 'corresponding author' on published papers create and link their Open Researcher and Contributor Identifier (ORCID) with their account on the Manuscript Tracking System (MTS), prior to acceptance. ORCID helps the scientific community achieve unambiguous attribution of all scholarly contributions. You can create and link your ORCID from the home page of the MTS by clicking on 'Modify my Springer Nature account'. For more information please visit please visit www.springernature.com/orcid.

Please do not hesitate to contact me if you have any questions or would like to discuss these revisions further.

We look forward to seeing the revised manuscript and thank you for the opportunity to review your work.

[Redacted]

Reviewer expertise:

Reviewer #1: evolution of sexual systems and sex chromosomes

Reviewer #2: evolution of sexual dimorphism, sex-biased gene expression

Reviewers' comments:

Reviewer #1 (Remarks to the Author):

I was referee #1 during the first round of reviews.

I am satisfied by some of the careful revisions made by the authors. Figures are now easier to understand. I find the new analysis on expression variability prior to sex biased expression evolution

very informative and well included in the new manuscript version. However, I insist on two previous problems I pointed out that the authors failed to solve (Figures 3a and 4a). Additionally, I carefully reread the manuscript and I realized there were a few additional points that I did not think about last time (with apologies) and that I believe should be controlled for before publication.

I still think there is an issue with Figure 4A (and Supplementary Figure S2), because for example some unbiased genes in Figure S2 show significant expression difference between males and females (In the S. pol-S. der pair, unbiased genes are significantly more expressed in males than females, which by definition should not be the case for unbiased genes). This is only an example but there are also issues for other pairs in Figures 4A and S2.

I also maintain that statistics should be paired in Figure 4A because a gene has its own intrisic expression level determined by its DNA sequence (as revealed by male and female expression correlation on Figure 1B), on top of that, male or female-specific effects can be added, causing in some cases sex-biased expression. Due to this male-female expression correlation, tests must be paired. For example, when comparing male to female expression in the S. pol-S. der pair, the t-test should be paired in R:

```
t.test(f, m, paired=TRUE)
```

where f is the vector of female expression averaged for each gene separately and m is the similar vector for male expression (genes must be in the same order in the m and f vectors).

About Figure 3a, previously I thought I did not understand it and asked for more information. But now I am almost certain there is an issue on this Figure because all ASO genes should also be DSO by definition. But on the figure ASO genes only share some points with DSO and with pairs of species.

The authors toned down their conclusion regarding convergence on sex-biased genes function. I am still not fully convinced this is a "considerable" convergence. I agree though that there does not seem to be statistics available to specifically test this.

After re-reading the manuscript, I realized that the affirmation that cosexual expression is closer to female expression is only true for sex-biased genes. Because when looking at all genes (Figure 4E), males and females are very similar and cosexuals very different. Maybe this point should be clarified in the text.

I realized that the lack of correlation between sexual dimorphism and the % of sex biased genes also indicates that sexual selection in not the main player in sex biased expression evolution, a point that maybe should be emphasized in the abstract and discussion.

Finally, there is a potential issue about Figure 4e that I did not realize last time. The 0 is missing from the x axis, please add it. It seems that cosexuals are centered around zero on both x and y axes of the PCA. This means that expression variation for cosexuals is not explained by axis 1 nor axis 2 of the PCA. This could be due to the fact that dioicous species have twice as many samples than cosexuals and drive the PCA axes. Could you please replot the PCA with only males and only females from dioicous species in order to have equal sample size between dioicous and monoicous? Does this modify the conclusion regarding convergent evolution of monoicous species expression? These control Figures may become supplementary.

Reviewer #2 (Remarks to the Author):

The authors have addressed my previous comments and I find that the clarity of the manuscript has been improved. However, I am still not convinced of the interpretation that sex-biased genes show a “considerable level” of functional convergence across the studied species. Given that this constitutes one of the main findings of this study, I think further clarification and revision is needed.

Lines 171-175: “We detected significant enrichment of GO terms for biological processes related to ‘microtubule’, ‘ion transport’ and ‘cilium’ consistently for male-biased genes. Conversely, the sets of female-biased genes of all or the majority of the studied species were enriched for GO terms related to ‘photosynthesis /chlorophyl processes’, ‘metabolism’ and ’oxidation reduction’ (Figure S1, Table S5).”

As far as I can tell from Fig. S1 and Table S5, a significant enrichment of “microtubule” for male-biased genes is only the case in one of the four species (S. polyschides). So, to include this term in the list of terms that show a consistent pattern for male-biased genes across the species seems to me a bit of a stretch. In relation to terms enriched across female-biased genes, only D. herbacea female-biased genes have an associated GO term related to ‘chlorophyl’, while ‘metabolism’ is extremely vague. “Carbohydrate metabolic process” is significantly enriched for both female-biased genes (in D. herbacea and S. firma) and male-biased genes (S. rigidula), “lipid metabolic process” is found across some female-biased genes (S. firma and S. polyschides), while “chitin metabolic process” is found only in male-biased genes (S. polyschides). So, the only clear association that I can see for female-biased genes is with GO terms related to ‘oxidation/reduction’.

Moreover, it is also not at all clear why these particular processes or GO terms would be expected to be enriched in one sex-biased gene category versus the other. How do these biological processes relate to sex-specific adaptation or sexual dimorphism?

********************END********************

Author Rebuttal, first revision:

Reviewers' comments:

Reviewer #1 (Remarks to the Author):

I was referee #1 during the first round of reviews.

I am satisfied by some of the careful revisions made by the authors. Figures are now easier to understand. I find the new analysis on expression variability prior to sex biased expression evolution very informative and well included in the new manuscript version. However, I insist on two previous problems I

pointed out that the authors failed to solve (Figures 3a and 4a). Additionally, I carefully reread the manuscript and I realized there were a few additional points that I did not think about last time (with apologies) and that I believe should be controlled for before publication.

Re: We sincerely thank the reviewer for the comments that helped improving the manuscript. We hope that our responses below solve all the concerns.

I still think there is an issue with Figure 4A (and Supplementary Figure S2), because for example some unbiased genes in Figure S2 show significant expression difference between males and females (In the S. pol-S. der pair, unbiased genes are significantly more expressed in males than females, which by definition should not be the case for unbiased genes). This is only an example but there are also issues for other pairs in Figures 4A and S2.

Re: We agree that the pattern highlighted by the reviewer in Figure S2 is not intuitive, but considering how DEseq2 identifies sex-biased genes independently of the TPM calculation using kallisto, this result is actually not unexpected. Indeed, Deseq2 takes as input a table of raw counts and performs an internal normalization where geometric mean is calculated for each gene across all samples. The counts for a gene in each sample is then divided by this mean. The median of these ratios in a sample is the size factor for that sample. This procedure corrects for library size and RNA composition bias, which can arise e.g. when only a small number of genes are very highly expressed in one experiment condition but not in the other. DESeq2 fits negative binomial generalized linear models for each gene and uses the Wald test for significance testing. The analysis output of Deseq2 consists of a table containing the significantly differentially expressed genes which can then be re-filtered by the user. In our manuscript, we used conservative filtering (Fold Change >2, padj <0.05) to assign bias to differential expressed genes (note that these thresholds are commonly used in the literature; e.g Lichilin et al Phil. Trans. R. Soc, 2021, Lipinska et al. Gen. Biol. 2017, 2019). Conversely, TPMs are not calculated by DEseq2 but by kallisto, which uses pseudo-alignments of RNAseq reads followed by conversion of counts into transcripts per million for normalization. Consequently, a gene can show a mean difference in TPM between males and females but be considered ‘unbiased’ by DEseq2 because of the filters/thresholds used. For example (please see new Table S3, where we include the output of DEseq2 together with the corresponding TPM values calculated by kallisto) gene g10015 (in *S. polyschides*) exhibits a difference in TPM between males and females (TPM female=3.56; TPM male=7.13) but this gene does not pass the filtering steps of DEseq2 so it is considered an ‘unbiased gene’.

In the case of sex biased genes in *S.rigidula* (Fig. 4A), FBG in males and females show low abundance (measured as log2TPM), which is close to significant when median expression are compared (p=0.07). However, as we discuss above, the TPMs are computed independently from Deseq2 differential expression analysis and are used to measure relative abundance of transcripts and global expression trends, rather then call significantly differentially expressed genes between samples. The statistical analysis by Deseq2 is robust to find differential expression in genes with low abundance and we trust that the FBG set predicted by Deseq2 is correct. Note that in the case of the MBG of S. rigidula, the relatively low number of genes (22) affects the statistical analysis.

In conclusion, given the conservative thresholds used for DEseq2, it is not surprising that genes that are considered ‘unbiased’ by DEseq2 still display some difference in expression between sexes, which

explains the pattern seen in Figure S2. Importantly, we would also like to stress that our study is focused on the patterns of sex-biased gene expression as inferred from DESeq2 and their expression fate after the transition to monoicy, so we believe that it is better to use a conservative approach in what we call 'sex-bias', and therefore our choice of strict threshold is acceptable.

Note that a similar pattern is also evident in other studies where TPMs of unbiased genes are plotted (e.g. Hachtett et al, Bioarchives 2021 and Ma et al, Genes 2020).

I also maintain that statistics should be paired in Figure 4A because a gene has its own intrinsic expression level determined by its DNA sequence (as revealed by male and female expression correlation on Figure 1B), on top of that, male or female-specific effects can be added, causing in some cases sex-biased expression. Due to this male-female expression correlation, tests must be paired. For example, when comparing male to female expression in the S. pol-S. der pair, the t-test should be paired in R:

```
t.test(f, m, paired=TRUE)
```

where f is the vector of female expression averaged for each gene separately and m is the similar vector for male expression (genes must be in the same order in the m and f vectors).

Re: We have followed the recommendations of the reviewer and editor and applied paired t-tests. The results remain unchanged except that there is an increase in the significance level of the comparison of expression between male and female expression of male-biased genes for the species pair S. rigidula-H. paniculata. Very importantly, this new statistical analysis does not affect in any way the conclusions of the paper.

About Figure 3a, previously I thought I did not understand it and asked for more information. But now I am almost certain there is an issue on this Figure because all ASO genes should also be DSO by definition.

But on the figure ASO genes only share some points with DSO and with pairs of species.

Re: We fully agree with the reviewer that Figure 3a is very difficult to interpreter. Not only the intercepts are complicated to read, but the confusion in the intersects between gene sets that are visible in the Fig3a (e.g not all ASOs being included in the DSOs) are caused by the handling of the different orthologous gene sets:

First, as explained in the results section of the manuscript, the single-copy orthologs list was 'supplemented' by the addition of 1:1:1:0 and 1:1:1:2 orthogroups for the DSOs and of 1:1:1:1:1:1:1:0 and 1:1:1:1:1:1:1:2 for the ASOs. This treatment allowed us to recover more genes for the analysis, as explained in the manuscript, but also lead to some DSOs not being included within the ASOs set. For example, if a set of DSOs was found to be 1:1:1:2, and the inference across all the 8 species found also two paralogs in a monoicous species, this would lead to the orthogroups containing 1:1:1:1:1:1:2:2 being excluded from the ASO set.

Secondly, the inference of orthogroups with Orthofinder among the four dioicous species (DSO) was performed independently from the inference of orthologs for all eight species (ASO). This methodological choice was justified by the fact that we wanted to focus on the evolution of sex-biased genes among the four dioicous species only, for building Fig. 3B (turnover of sex-biased genes). Considering that we had

sufficient species sampling using only the dioicous species (based on orthofinder best practices) this more targeted approach improved our ortholog recall in orthofinder (Emms and Kelly Gen. Biol. 2019).

An alternative method could have been to infer the different gene sets from a single, global Orthofinder run. This method retrieves less DSOs, but gives fundamentally similar results compared to the method we used in the manuscript (Reviewer's Figure 1): highly branch-specific identity of sex-biased genes, no ortholog with shared sex-bias across all dioicous species (as expected by chance), 38 ancestrally sex-biased orthogroups, and a PIC (when measured in species where orthologous genes are unbiased) significantly different from that of unbiased genes. In summary, the conclusions are no different regardless we use independent orthofinder runs or one orthofinder run, but the advantage of using independent runs is that we retrieve more OGs for our DSO analysis (with the disadvantage being that the intercepts Figure 3a are counterintuitive). We have not reported these extra-analyses on the manuscript for a matter of simplicity, but we can include this information as a supplemental material if necessary.

In conclusion, Figure 3a is not 'wrong', but we fully agree it is misleading, and we thank the reviewer for having noticed that it brings confusion. Note that the list and numbers of genes in each orthologous set is already reported in the supplemental tables, so in retrospect, we should not have included it as main figure because it does not add any major information. Therefore, we propose to transfer it to the supplementary figures (Figure S1 in the new version of the manuscript) clearly indicating in the legend how each set of orthologs was generated.

The authors toned down their conclusion regarding convergence on sex-biased genes function. I am still not fully convinced this is a "considerable" convergence. I agree though that there does not seem to be statistics available to specifically test this.

Re: We have removed the word 'considerable' and rephrased the sentences related to this conclusion in order to further tone-down this point.

After re-reading the manuscript, I realized that the affirmation that cosexual expression is closer to female expression is only true for sex-biased genes. Because when looking at all genes (Figure 4E), males and females are very similar and cosexuals very different. Maybe this point should be clarified in the text.

Re: Indeed, Figure 4E shows that (not surprisingly) there is a closer relationship in expression within species rather than between species, i.e., males and females of the same species are closer on the PCA plot. The figure aims to highlight the pattern of grouping of monoicous species together (instead of by phylogenetic distance), illustrating the convergence of gene expression during the transition from dioicy towards monoicy.

The reviewer is correct that the fact that female expression is closer to monoicous expression is mainly driven by sex-biased genes, as we state in lines 251-254: "Taken together, the above observations suggest that gene expression profiles of monoicous species tend to be more closely related to the females of the related dioicous species, and this similarity appears to be driven by sex-biased genes, in particular male-biased genes."

Furthermore, note that when all genes are included (sex biased and unbiased) we observe that females consistently have closer expression profiles to the co-sexual counterparts compared with males (Figure

S5). The co-sexual expression is therefore closer to females than to males (but of course male and females of the same species are the closest). This pattern is mainly driven by the sex-biased genes, as shown by Pearson coefficient analysis in Figure 4B.

These observations are described in the results section: "Interestingly, with the exception of the Ectocarpales species pair (*S. firma*-*C. linearis*), sex-biased gene expression profiles diverged significantly less from the monoicous species than did that of the unbiased genes (Figure 4B, S5). Overall, the expression profile similarity observed between females and monoicous individuals were mainly driven by expression patterns of male-biased genes".

I realized that the lack of correlation between sexual dimorphism and the % of sex biased genes also indicates that sexual selection in not the main player in sex biased expression evolution, a point that maybe should be emphasized in the abstract and discussion.

Re: The reviewer is correct, and although we had discussed this point in the discussion section it is indeed a good idea to make it clearer. We propose to rephrase the paragraph as follows:

"Thus, it would be expected that species showing more prominent differences in morphology between male and female would also be characterized by high levels of sex-biased gene expression, as has been shown to be the case in birds [37]. Our study, in contrast, revealed no correlation between the level of sex-biased gene expression and the degree of phenotypic sexual dimorphism in the brown algae studied here. Therefore, the link between gene expression evolution and sexual selection is uncertain for these organisms, and sexual selection is likely not to be the main driver of the sex-biased gene expression evolution. This observation may reflect a lower degree of sexual selection in the brown algae compared with animals."

Finally, there is a potential issue about Figure 4e that I did not realize last time. The 0 is missing from the x axis, please add it. It seems that cosexuals are centered around zero on both x and y axes of the PCA. This means that expression variation for cosexuals is not explained by axis 1 nor axis 2 of the PCA. This could be due to the fact that dioicous species have twice as many samples than cosexuals and drive the PCA axes. Could you please replot the PCA with only males and only females from dioicous species in order to have equal sample size between dioicous and monoicous? Does this modify the conclusion regarding convergent evolution of monoicous species expression? These control Figures may become supplementary.

Re: We have added the x axis numbers, this was indeed a typo. We thank the reviewer for proposing this analysis. As requested, we have generated PCA plots including only males and only females, as well as a mix of males and females from dioicous species, taken randomly, with a similar number of samples as in the monoicous species. We observe that the tendencies reported in our paper are maintained on these plots and that the number of samples does not seem to drive the patterns described in the manuscript (Reviewer Figure 2).

Most importantly, note that the level of convergence in gene expression is visible on the PCA plot, but the assessment of convergence is based on independent statistical tests (including likelihood ratio tests on the effect of sexual system on expression levels across single-copy orthologs, described in the section

Materials and Methods). To conclude, there is no change of the conclusions of the manuscript regarding convergent evolution of monoicous species expression.

(Note that if the editor and reviewer request, Reviewer Figure 2 can be included in the manuscript as a supplemental figure).

Reviewer #2 (Remarks to the Author):

The authors have addressed my previous comments and I find that the clarity of the manuscript has been improved. However, I am still not convinced of the interpretation that sex-biased genes show a “considerable level” of functional convergence across the studied species. Given that this constitutes one of the main findings of this study, I think further clarification and revision is needed.

Re: We believe our data clearly shows that there are at least some shared GO terms across the SBG in each of the different species, and that given that these species do not share the identity of the sex biased genes, we believe that it is important to report this finding. Moreover, the functions that are shared among dioicous species may be associated to sex-specific biological functions (see below). However, we agree with the reviewer that we should tone down this conclusion because of the impossibility of establishing solid statistical tests and we should avoid being utterly speculative. We have therefore further modified the general conclusion of this paragraph (L217) with the following sentence: “Taken together, our results indicate that whilst sex-biased genes exhibited a high turnover rate during brown alga diversification, some of their predicted functions were conserved across dioicous species.” Along these lines, we also further toned down the abstract with the following sentence: “Although sex-biased genes exhibited a high turnover rate during brown alga diversification, some of their predicted functions were conserved across species”. We have also added a sentence in the discussion to link the putative function of SBG to sex-specific biology (see below).

Lines 171-175: “We detected significant enrichment of GO terms for biological processes related to ‘microtubule’, ‘ion transport’ and ‘cilium’ consistently for male-biased genes. Conversely, the sets of female-biased genes of all or the majority of the studied species were enriched for GO terms related to ‘photosynthesis /chlorophyl processes’, ‘metabolism’ and ’oxidation reduction’ (Figure S1, Table S5).” As far as I can tell from Fig. S1 and Table S5, a significant enrichment of “microtubule” for male-biased genes is only the case in one of the four species (S. polyschides). So, to include this term in the list of terms that show a consistent pattern for male-biased genes across the species seems to me a bit of a stretch.

In relation to terms enriched across female-biased genes, only D. herbacea female-biased genes have an associated GO term related to ‘chlorophyl’, while ‘metabolism’ is extremely vague. “Carbohydrate metabolic process” is significantly enriched for both female-biased genes (in D. herbacea and S. firma) and male-biased genes (S. rigidula), “lipid metabolic process” is found across some female-biased genes (S. firma and S. polyschides), while “chitin metabolic process” is found only in male-biased genes (S. polyschides). So, the only clear association that I can see for female-biased genes is with GO terms related to ‘oxidation/reduction’.

Re: We removed the term ‘microtubule’ from the text, the reviewer is correct that indeed it should not have been included in the list of terms. The corrected text is now: “We detected significant enrichment of GO terms for biological processes related to ‘ion- and transmembrane transport’ and ‘cilium’ often associated to male-biased genes across dioicous species. Conversely, the sets of female-biased genes of all the studied species were enriched for GO terms related to oxidation/reduction (Figure S2, Table S6).”

Note that in three out of four species there is enrichment in photosynthesis processes (chlorophyl, photosystems, etc), so we assumed that there is an association with photosynthesis. But we decided to follow the reviewer advice and left out this term and mention only oxidation-reduction processes in the text of the revised manuscript. The terms that are enriched in more than 2/4 species are highlighted in bold in Table S6.

Moreover, it is also not at all clear why these particular processes or GO terms would be expected to be enriched in one sex-biased gene category versus the other. How do these biological processes relate to sex-specific adaptation or sexual dimorphism?

Re: We thank the reviewer for this interesting comment. Indeed, in most algal species, female gametophytes usually grow faster and bigger (Luthringer et al. MBE 2015; Luthringer et al. PIP 2017), therefore more oxidative metabolism may be related to this faster and more conspicuous growth. Male gametophytes, conversely, are smaller and produce many flagellated gametes that swim for longer in order to reach the female gamete (which produces a sperm-attracting pheromone). Moreover, pheromone produced by females comprise linear or alicyclic unsaturated hydrocarbons derived from the aliphatic terminus of C(20) polyunsaturated fatty acids (PUFAs) by oxidative cleavage (Rui and Boland, J. Org. Chem, 2010), which may, again, explain increased oxidative metabolism in females. Cilia and flagella are fundamental units of motion in cellular biology both being hair-like organelles sharing a common basic structure. The terms ‘cilia’ and ‘flagella’ are often used interchangeably, because there is no intrinsic structural distinction between them. ‘flagella’ typically refers to sparse or isolated structures used by unicellular organisms for locomotion (like in the case of male gametes of brown algae), whereas ‘cilia’ refers to extended groups occurring in protozoans and animals (Gilpin et al Nature 2020). Flagellar rotation (at least in bacteria) requires ion-motive force (IMF), the electro-chemical potential difference built across the membrane so that flagella movement is driven by the flux of ions across the membrane (Larsen et al., PNAS 1974; Manson et al., PNAS 1977).

Therefore, overall, the terms that are highlighted as male and female enriched do appear to have some link to the sex-specific biological processes. We have added a sentence mentioning the link between sex-specific biology and SBG functional enrichment in the discussion section, without entering in too much detail to avoid being too speculative.

“(…) These functions may be associated with sex-specific biological processes. For example, the more conspicuous growth of female gametophytes producing larger gametes which secrete a sperm-attracting pheromone and, conversely, production of fast-swimming bi-flagellated sperm by the male gametophyte.” (lines 371-374).

References

Emms, DM and Kelly,S (2019). OrthoFinder: phylogenetic orthology inference for comparative genomics. Genome Biol 20, 238

Hatchett et al. (2021) - Evolutionary dynamics of sex-biased gene expression in a young XY system: insights from the brown algae. bioRxiv 2021.08.12.455804 doi:https://doi.org/10.1101/2021.08.12.455804

Gilpin, W., Bull, M.S. & Prakash, M. (2020). The multiscale physics of cilia and flagella. Nat Rev Phys 2, 74–88 https://doi.org/10.1038/s42254-019-0129-0

Larsen, S. H., Adler, J., Gargus, J. J., and Hogg, R. W. (1974). Chemomechanical coupling without ATP: the source of energy for motility and chemotaxis in bacteria. Proc. Natl. Acad. Sci. U.S.A. 71, 1239–1243. doi: 10.1073/pnas.71.4.1239

Lichilín N., El Taher A and Böhne A. (2021) Sex-biased gene expression and recent sex chromosome turnover. Phil. Trans. R. Soc. B 376: 20200107.20200107

Lipinska A, Toda N, Heesch S, Peters A, Cock JM, Coelho SM (2017). Multiple gene movements into and out of haploid sex chromosomes. 2017 June, Genome Biology , doi: 10.1186/s13059-017-1201-7

Lipinska AP, Serrano-Serrano M., Cormier A., Peters AF, Kogame K, Cock JM, Coelho SM (2018). Rapid turnover of life-cycle-related genes in the brown algae. Genome Biology, doi: 10.1186/s13059-019-1630-6

Luthringer R, Cormier A, Ahmed S, Peters, AF, Cock JM, Coelho SM (2014) Sexual dimorphism in the brown algae. Perspectives in Phycology 1: 11-25.

Luthringer R, Lipinska A, Roze D, Cormier A, Peters A, Cock JM, Coelho SM (2015) The pseudoautosomal regions of the U/V sex chromosomes of the brown alga Ectocarpus exhibit unusual features. Mol Biol Evol. doi: 10.1093/molbev/msv173 Luthringer PIP

Ma, W. J., Veltsos, P., Toups, M. A., Rodrigues, N., Sermier, R., Jeffries, D. L., & Perrin, N. (2018). Tissue Specificity and Dynamics of Sex-Biased Gene Expression in a Common Frog Population with Differentiated, Yet Homomorphic, Sex Chromosomes. Genes, 9(6), 294.

Manson, M. D., Tedesco, P., Berg, H. C., Harold, F. M., and Van der Drift, C. (1977). A protonmotive force drives bacterial flagella. Proc. Natl. Acad. Sci. U.S.A. 74, 3060–3064. doi: 10.1073/pnas.74.7.3060

Rui and Boland (2010) Algal Pheromone Biosynthesis: Stereochemical Analysis and Mechanistic Implications in Gametes of Ectocarpus siliculosus. The Journal of Organic Chemistry 75(12):3958-64 DOI:10.1021/jo1004372

Decision Letter, second revision:

4th January 2022

Dear Dr. Coelho,

Thank you for submitting your revised manuscript "SELECTION DRIVES THE EVOLUTION OF CONVERGENT GENE EXPRESSION CHANGES DURING TRANSITIONS TO CO-SEXUALITY IN HAPLOID SEXUAL SYSTEMS" (NATECOLEVOL-210613874B). It has now been seen again by the original reviewers and their comments are below. The reviewers find that the paper has improved in revision, and therefore we'll be happy in principle to publish it in Nature Ecology & Evolution, pending minor revisions to satisfy the reviewers' final requests and to comply with our editorial and formatting guidelines.

If the current version of your manuscript is in a PDF format, please email us a copy of the file in an editable format (Microsoft Word or LaTex)-- we can not proceed with PDFs at this stage.

We are now performing detailed checks on your paper and will send you a checklist detailing our editorial and formatting requirements in about a week. Please do not upload the final materials and make any revisions until you receive this additional information from us.

Thank you again for your interest in Nature Ecology & Evolution. Please do not hesitate to contact me if you have any questions.

[REDACTED]

Reviewer #1 (Remarks to the Author):

I would like to thank the authors for their detailed answers and additional analyses. I am now fully satisfied with the manuscript.
I agree new Figure S1 is best as supplement.
I leave it up to the authors and editor to decide whether Reviewer Figure 2 should be included as Supplementary, I am glad the pattern remains in this control and it might not be necessary to include this Figure in the final manuscript.

Reviewer #2 (Remarks to the Author):

The authors have addressed all of my comments and I think the manuscript is suitable for publication. I do not have any further remarks.

Our ref: NATECOLEVOL-210613874B

18th January 2022

Dear Dr. coelho,

Thank you for your patience as we've prepared the guidelines for final submission of your Nature Ecology & Evolution manuscript, "SELECTION DRIVES THE EVOLUTION OF CONVERGENT GENE EXPRESSION CHANGES DURING TRANSITIONS TO CO-SEXUALITY IN HAPLOID SEXUAL SYSTEMS" (NATECOLEVOL-210613874B). Please carefully follow the step-by-step instructions provided in the attached file, and add a response in each row of the table to indicate the changes that you have made. Please also check and comment on any additional marked-up edits we have proposed within the text. Ensuring that each point is addressed will help to ensure that your revised manuscript can be swiftly handed over to our production team.

We would like to start working on your revised paper, with all of the requested files and forms, as soon as possible (preferably within two weeks). Please get in contact with us immediately if you anticipate it taking more than two weeks to submit these revised files.

When you upload your final materials, please include a point-by-point response to any remaining reviewer comments.

If you have not done so already, please alert us to any related manuscripts from your group that are under consideration or in press at other journals, or are being written up for submission to other journals (see: https://www.nature.com/nature-research/editorial-policies/plagiarism#policy-on-duplicate-publication for details).

In recognition of the time and expertise our reviewers provide to Nature Ecology & Evolution's editorial process, we would like to formally acknowledge their contribution to the external peer review of your manuscript entitled "SELECTION DRIVES THE EVOLUTION OF CONVERGENT GENE EXPRESSION CHANGES DURING TRANSITIONS TO CO-SEXUALITY IN HAPLOID SEXUAL SYSTEMS". For those reviewers who give their assent, we will be publishing their names alongside the published article.

Nature Ecology & Evolution offers a Transparent Peer Review option for new original research manuscripts submitted after December 1st, 2019. As part of this initiative, we encourage our authors to support increased transparency into the peer review process by agreeing to have the reviewer comments, author rebuttal letters, and editorial decision letters published as a Supplementary item. When you submit your final files please clearly state in your cover letter whether or not you would like to participate in this initiative. Please note that failure to state your preference will result in delays in accepting your manuscript for publication.

Cover suggestions

As you prepare your final files we encourage you to consider whether you have any images or illustrations that may be appropriate for use on the cover of Nature Ecology & Evolution.

Covers should be both aesthetically appealing and scientifically relevant, and should be supplied at the best quality available. Due to the prominence of these images, we do not generally select images featuring faces, children, text, graphs, schematic drawings, or collages on our covers.

We accept TIFF, JPEG, PNG or PSD file formats (a layered PSD file would be ideal), and the image should be at least 300ppi resolution (preferably 600-1200 ppi), in CMYK colour mode.

If your image is selected, we may also use it on the journal website as a banner image, and may need to make artistic alterations to fit our journal style.

Please submit your suggestions, clearly labeled, along with your final files. We'll be in touch if more information is needed.

Nature Ecology & Evolution has now transitioned to a unified Rights Collection system which will allow our Author Services team to quickly and easily collect the rights and permissions required to publish your work. Approximately 10 days after your paper is formally accepted, you will receive an email in providing you with a link to complete the grant of rights. If your paper is eligible for Open Access, our Author Services team will also be in touch regarding any additional information that may be required to arrange payment for your article.

Please note that <i>Nature Ecology & Evolution</i> is a Transformative Journal (TJ). Authors may publish their research with us through the traditional subscription access route or make their paper immediately open access through payment of an article-processing charge (APC). Authors will not be required to make a final decision about access to their article until it has been accepted. Find out more about Transformative Journals

<B>Authors may need to take specific actions to achieve compliance with funder and institutional open access mandates. For submissions from January 2021, if your research is supported by a funder that requires immediate open access (e.g. according to Plan S principles) then you should select the gold OA route, and we will direct you to the compliant route where possible. For authors selecting the subscription publication route our standard licensing terms will need to be accepted, including our self-archiving policies. Those standard licensing terms will supersede any other terms that the author or any third party may assert apply to any version of the manuscript.

Please note that you will not receive your proofs until the publishing agreement has been received through our system.

For information regarding our different publishing models please see our Transformative Journals page. If you have any questions about costs, Open Access requirements, or our legal forms, please contact ASJournals@springernature.com.

Please use the following link for uploading these materials:

[REDACTED]

If you have any further questions, please feel free to contact me.

[REDACTED]

Reviewer #1:
Remarks to the Author:
I would like to thank the authors for their detailed answers and additional analyses. I am now fully satisfied with the manuscript.
I agree new Figure S1 is best as supplement.
I leave it up to the authors and editor to decide whether Reviewer Figure 2 should be included as Supplementary, I am glad the pattern remains in this control and it might not be necessary to include this Figure in the final manuscript.

Reviewer #2:
Remarks to the Author:
The authors have addressed all of my comments and I think the manuscript is suitable for publication. I do not have any further remarks.

Final Decision Letter:

7th February 2022

Dear Dr coelho,

We are pleased to inform you that your Article entitled "SELECTION DRIVES CONVERGENT GENE EXPRESSION CHANGES DURING TRANSITIONS TO CO-SEXUALITY IN HAPLOID SEXUAL SYSTEMS", has now been accepted for publication in Nature Ecology & Evolution.

Over the next few weeks, your paper will be copyedited to ensure that it conforms to Nature Ecology and Evolution style. Once your paper is typeset, you will receive an email with a link to choose the appropriate publishing options for your paper and our Author Services team will be in touch regarding any additional information that may be required

After the grant of rights is completed, you will receive a link to your electronic proof via email with a request to make any corrections within 48 hours. If, when you receive your proof, you cannot meet this deadline, please inform us at rjsproduction@springernature.com immediately.

You will not receive your proofs until the publishing agreement has been received through our system

Due to the importance of these deadlines, we ask you please us know now whether you will be difficult to contact over the next month. If this is the case, we ask you provide us with the contact information (email, phone and fax) of someone who will be able to check the proofs on your behalf, and who will

be available to address any last-minute problems . Once your paper has been scheduled for online publication, the Nature press office will be in touch to confirm the details.

Acceptance of your manuscript is conditional on all authors' agreement with our publication policies (see www.nature.com/authors/policies/index.html). In particular your manuscript must not be published elsewhere and there must be no announcement of the work to any media outlet until the publication date (the day on which it is uploaded onto our web site).

Please note that <i>Nature Ecology & Evolution</i> is a Transformative Journal (TJ). Authors may publish their research with us through the traditional subscription access route or make their paper immediately open access through payment of an article-processing charge (APC). Authors will not be required to make a final decision about access to their article until it has been accepted. Find out more about Transformative Journals

<B>Authors may need to take specific actions to achieve compliance with funder and institutional open access mandates. For submissions from January 2021, if your research is supported by a funder that requires immediate open access (e.g. according to Plan S principles) then you should select the gold OA route, and we will direct you to the compliant route where possible. For authors selecting the subscription publication route our standard licensing terms will need to be accepted, including our self-archiving policies. Those standard licensing terms will supersede any other terms that the author or any third party may assert apply to any version of the manuscript.

In approximately 10 business days you will receive an email with a link to choose the appropriate publishing options for your paper and our Author Services team will be in touch regarding any additional information that may be required.

You will not receive your proofs until the publishing agreement has been received through our system.

If you have any questions about our publishing options, costs, Open Access requirements, or our legal forms, please contact ASJournals@springernature.com

An online order form for reprints of your paper is available at https://www.nature.com/reprints/author-reprints.html. All co-authors, authors' institutions and authors' funding agencies can order reprints using the form appropriate to their geographical region.

We welcome the submission of potential cover material (including a short caption of around 40 words) related to your manuscript; suggestions should be sent to Nature Ecology & Evolution as electronic files (the image should be 300 dpi at 210 x 297 mm in either TIFF or JPEG format). Please note that such pictures should be selected more for their aesthetic appeal than for their scientific content, and that colour images work better than black and white or grayscale images. Please do not try to design a cover with the Nature Ecology & Evolution logo etc., and please do not submit composites of images

related to your work. I am sure you will understand that we cannot make any promise as to whether any of your suggestions might be selected for the cover of the journal.

You can now use a single sign-on for all your accounts, view the status of all your manuscript submissions and reviews, access usage statistics for your published articles and download a record of your refereeing activity for the Nature journals.

To assist our authors in disseminating their research to the broader community, our SharedIt initiative provides you with a unique shareable link that will allow anyone (with or without a subscription) to read the published article. Recipients of the link with a subscription will also be able to download and print the PDF.

You can generate the link yourself when you receive your article DOI by entering it here: <a href="http://authors.springernature.com/share">http://authors.springernature.com/share<a>.

[REDACTED]

P.S. Click on the following link if you would like to recommend Nature Ecology & Evolution to your librarian http://www.nature.com/subscriptions/recommend.html#forms

** Visit the Springer Nature Editorial and Publishing website at www.springernature.com/editorial-and-publishing-jobs for more information about our career opportunities. If you have any questions please click here.**
